# Loss of the mitochondrial protein SPD-3 elevates PLK-1 levels and dysregulates mitotic events

**DOI:** 10.26508/lsa.202302011

**Published:** 2023-09-08

**Authors:** Yu-Zen Chen, Vitaly Zimyanin, Stefanie Redemann

**Affiliations:** 1 Center for Membrane and Cell Physiology, School of Medicine, University of Virginia , Charlottesville, VA, USA; 2 Department of Molecular Physiology and Biological Physics, School of Medicine, University of Virginia , Charlottesville, VA, USA; 3 Department of Cell Biology, School of Medicine, University of Virginia , Charlottesville, VA, USA

## Abstract

In metazoans, Polo-like kinase (PLK1) controls several mitotic events including nuclear envelope breakdown, centrosome maturation, spindle assembly and progression through mitosis. Here we show that a mutation in the mitochondria-localized protein SPD-3 affects mitotic events by inducing elevated levels of PLK-1 in early *Caenorhabditis elegans* embryos. SPD-3 mutant embryos contain abnormally positioned mitotic chromosomes, show a delay in anaphase onset and asymmetrically disassemble the nuclear lamina. We found that more PLK-1 accumulated on centrosomes, nuclear envelope, nucleoplasm, and chromatin before NEBD, suggesting that PLK-1 overexpression is responsible for some of the observed mitotic phenotypes. In agreement with this, the chromosome positioning defects of the *spd-3(oj35)* mutant could be rescued by reducing PLK-1 levels. Our data suggests that the mitochondrial SPD-3 protein affects chromosome positioning and nuclear envelope integrity by up-regulating the endogenous levels of PLK-1 during early embryogenesis in *C. elegans*. This finding suggests a novel link between mitochondria and nuclear envelope dynamics and chromosome positioning by increasing the amount of a key mitotic regulator, PLK-1, providing a novel link between mitochondria and mitosis.

## Abstract (precis)

In *C. elegans* a mutation in the mitochondria-localized protein SPD-3 leads to elevated levels of Polo-like kinase 1, causing abnormal chromosome positioning during mitosis.

## Introduction

Mitochondria are organelles in which cellular respiration occurs, and a variety of other biosynthetic processes, such as the regulation of Ca^2+^, apoptosis, and the production of reactive oxygen species (Jeong & Seol, 2008; Tilokani et al, 2018; Amorim et al, 2022). Artificially disrupting mitochondrial function in oocytes and early embryos was shown to cause defects in maturation (Al-Zubaidi et al, 2021), fertilization (Pasquariello et al, 2019), errors in cell division and chromosome segregation (Mikwar et al, 2020; Wang et al, 2020), and loss of viability and defects in metabolism (Al-Zubaidi et al, 2021). These observations suggest a link between mitochondrial defects, spindle assembly abnormalities, chromosome segregation errors, and aneuploidy. As mitochondria are strongly involved in metabolism and the main generators of energy in form of ATP it is possible that changes in the available energy can negatively affect spindles. In particular, the function of motor proteins, which are required for spindle assembly and chromosome segregation depends on available ATP (Sweeney & Holzbaur, 2018), and changes in the ATP content could impair motor function, ultimately leading to mitotic errors (Cheng et al, 2021). However, the mechanisms of how defects in mitochondria affect spindle function in mitosis have remained elusive.

The *Caenorhabditis elegans* spindle defect-3 (SPD-3) protein is a mitochondria-localizing protein, which is only conserved in nematodes that was isolated in an ethyl methanesulfonate-mutagenesis screen in search of cell division mutants (O’Connell et al, 1998). Previous publications have shown that defects in the SPD-3 protein in the nematode *C. elegans* impair homolog pairing by causing a severe reduction in the mobility of SUN-1 aggregates at the start of meiotic prophase (Labrador et al, 2013). It was suggested that this defect is due to the reduced function of cytoskeletal motors in *spd-3* mutants, caused by a defect in mitochondrial function and associated changes in ATP levels (Labrador et al, 2013). This was further supported by the observation of mitotic spindle positioning defects in one-cell *C. elegans spd-3* mutant embryos, which are comparable to spindle positioning defects after dynein depletion (Yoder & Han, 2001; Dinkelmann et al, 2007).

Here, we show that mutation of *spd-3* does not only impair spindle positioning as previously reported but also chromosome positioning and the nuclear integrity during mitosis. Interestingly, we found that Polo-like kinase (PLK-1) levels are significantly increased in the *spd-3* mutant. PLK-1 is a crucial mitotic kinase that functions in many aspects of cell division, such as centrosome maturation (Fu et al, 2015), spindle assembly checkpoint (SAC) activity (Arnaud et al, 1998; Houston et al, 2023; Taylor et al, 2023), mitotic entry (Thomas et al, 2016), centrosome maturation (Fu et al, 2015), nuclear envelope (NE) disassembly (Dawson & Wente, 2017; Linder et al, 2017; Martino et al, 2017), and asymmetric cell division (Han et al, 2018) in *C. elegans* and mammalian cells.

PLK1 function is not limited to mitotic events and cytokinesis. PLK1 also regulates heat-shock transcription factor 1 (Kim et al, 2005), p53 (Ando et al, 2004) microtubule dynamics (Joukov & de Nicolo, 2018), and mitochondrial Ca^2+^ homeostasis (Lee et al, 2016). The tight regulation of PLK1 during the cell cycle is essential to mitotic progression and increased levels of PLK1 have been associated with cancer. Increasing evidence indicates that PLK1 overexpression correlates with poor clinical outcomes (Martini et al, 2019). Our data suggest that elevated PLK-1 in early *C. elegans* embryos leads to a delay in anaphase onset, asymmetric nuclear disassembly, and premature centriole splitting and could thus account for the observed abnormal mitosis. This finding provides a key link between mitochondria and mitosis by affecting master regulators of mitosis.

## Results

### The mitochondrial protein SPD-3 is required for mitotic events

**Figure S1.**
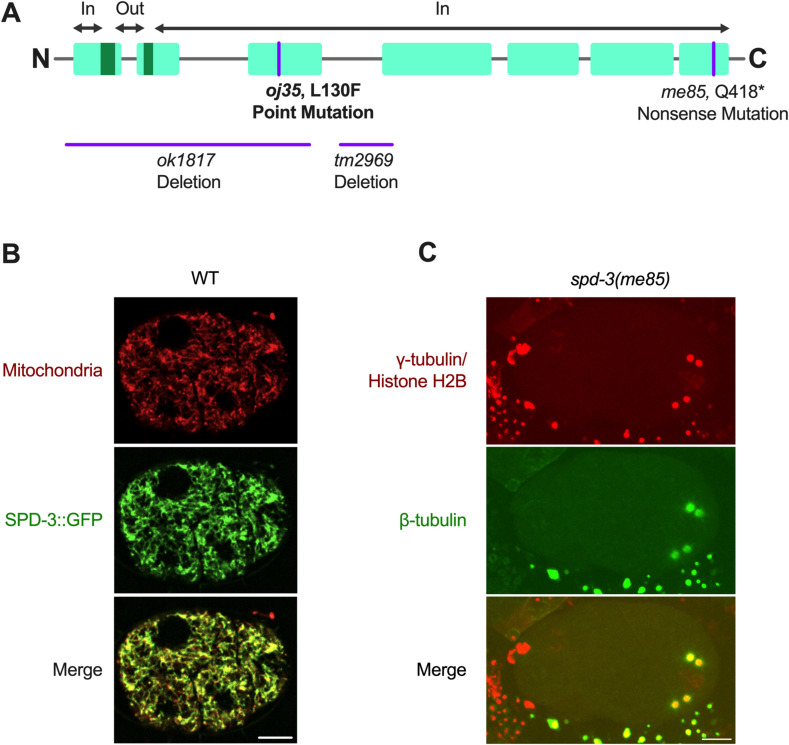
SPD-3 localizes to mitochondria, related to Fig 1. **(A)** Diagram of the *spd-3* gene indicating the position of the *oj35* and *me85* mutation, and two deletion alleles, *ok1817* and *tm2969*. The TOPCONS program predicted the putative transmembrane sequences of SPD-3. The term “in” denotes the location inside the mitochondria membrane, whereas “out” signifies the location outside the mitochondria membrane. **(B)** SPD-3::GFP colocalizes with mitochondria stained by the mitochondrial dye MitoTracker CMXRosetta in WT embryos. **(C)** Image of *spd-3(me85)* one-cell stage embryo expressing GFP::β-tubulin, mCherry::γ-tubulin, and mCherry::histone H2B. **(B, C)** Scale bar, 10 μm.

To further determine the functions of the mitochondrial protein SPD-3 during mitosis, we characterized the *spd-3(oj35)* mutant, which was previously shown to impact spindle alignment (Dinkelmann et al, 2007). The *spd-3(oj35)* mutant was isolated in an ethyl methanesulfonate mutagenesis screen in search of cell division mutants and carries a single cytosine-to-thymidine transition, resulting in a leucine-to-phenylalanine change at amino acid 130 (O’Connell et al, 1998; Dinkelmann et al, 2007). The *spd-3(oj35)* strain is a temperature-sensitive, maternal-effect mutant, which is defective in meiosis (Labrador et al, 2013) and mitosis (O’Connell et al, 1998; Dinkelmann et al, 2007). In addition to the *spd-3(oj35)* mutant, three additional mutant strains are available (Fig S1A). Two deletion alleles, *spd-3(ok1817)* and *spd-3(tm2969)*, are null *spd-3* alleles (Labrador et al, 2013) and are very sick. The third mutant strain *spd-3(me85)* carries an early stop mutation and has severe meiotic defects (Fig S1C, Video 1) impeding the analysis of mitosis (Labrador et al, 2013). Thus, we decided to focus on the effects of *spd-3* mutation in the *spd-3(oj35)* strain.

Video 1 Live-cell imaging of WT and *spd-3(me85)* embryos during mitosis. The WT, (left) and *spd-3(me85)* (right) embryos coexpressing GFP::β-tubulin, mCherry::γ-tubulin, and mCherry::histone H2B. Fluorescence confocal images were acquired every 15 s by collecting 17 z-planes at 1.0-μm intervals. Sale bar represents 10 μm.Download video

**Figure S2.**
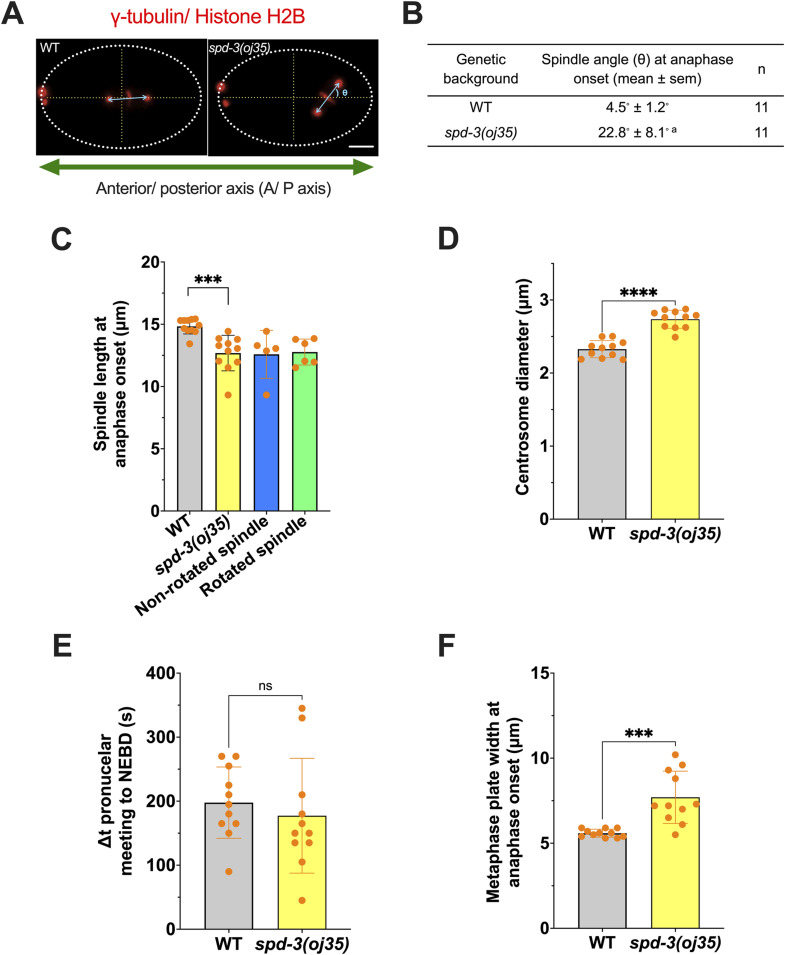
*spd-3(oj35)* is defective in mitotic spindle alignment and chromosome positioning, related to Fig 1. **(A)** Images of embryos coexpressing mCherry::histone H2B and mCherry::γ-tubulin in WT (left) and *spd-3(oj35)* (right). In WT embryos, the mitotic spindle is aligned along the anterior/posterior axis (A/P axis) at anaphase onset. The mitotic spindle failed to align to the A/P axis in *spd-3(j35)* embryos. The angle (θ) of the mitotic spindle in relation to the A/P axis is determined. Scale bars, 10 μm. **(B)** Table depicting spindle angle (θ) in WT (n = 11) and *spd-3(oj35)* (n = 11) embryos at anaphase onset. An “a” indicates *P < 0.05 in comparison to WT. **(C)** Graph plotting mitotic spindle length (pole-to-pole distance) at anaphase onset in WT (gray bar) and *spd-3(oj35)* (yellow bar). The spindle length of the non-rotated spindle is represented by the blue bar, whereas the green bar indicates the spindle length of the rotated spindle in *spd-3(oj35)*. **(D)** Plots of the centrosome size in WT (n = 11) and *spd-3(oj35)* (n = 11). The diameter of centrosomes was measured by the intensity of mCherry::γ-tubulin. **(E)** Plot of the duration of mitotic prophase. Duration of prophase is defined by the time between pronuclei meeting and NEBD. **(F)** Plot of the width of the metaphase plate at anaphase onset. Error bars are SD. N = 11 embryos for WT and *spd-3(oj35)*. The significance of differences between results was determined by two-tailed *t* tests (ns, not significant difference; ***P < 0.001).

**Figure 1.**
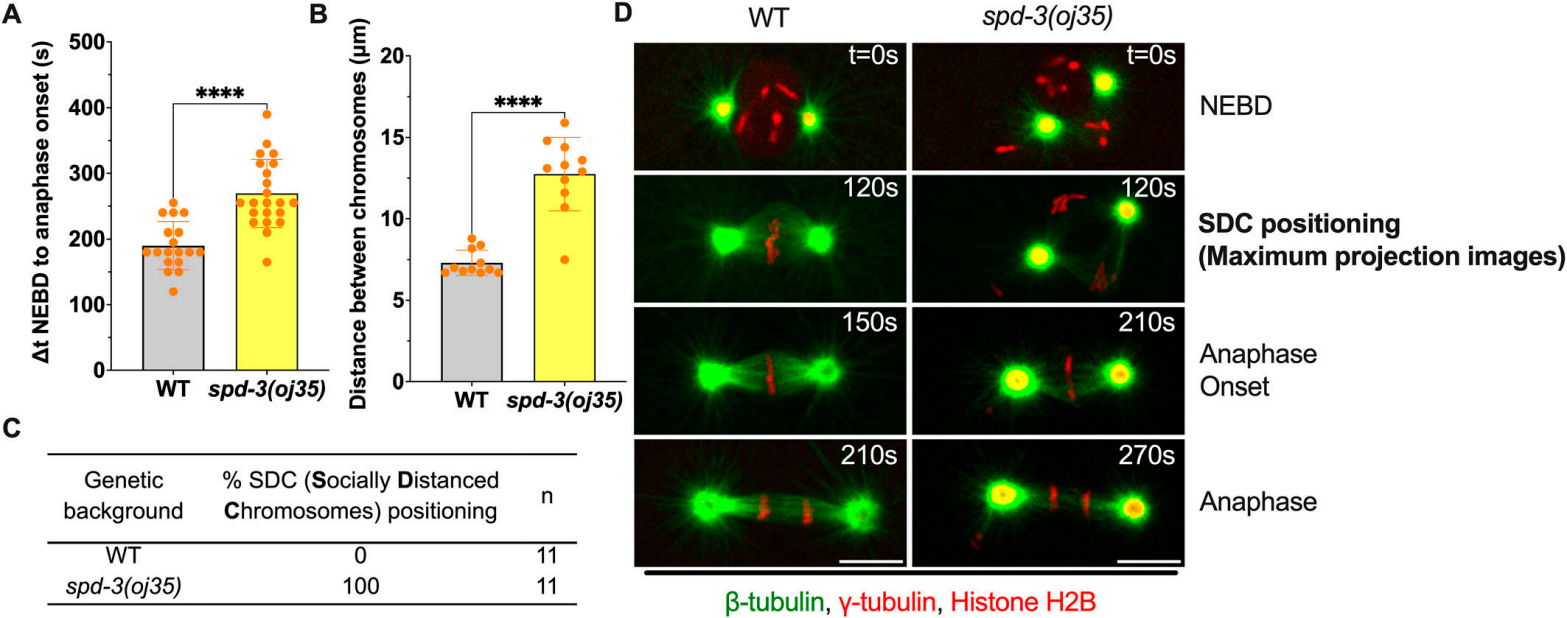
Duration of mitosis and chromosome positioning in *spd-3(oj35)* one-cell stage *C. elegans* embryos. **(A)** Graph plotting the duration of time between NEBD and anaphase onset in WT, (gray bar) and *spd-3(oj35)* (yellow bar). **(B)** Plot of the maximum distance between chromosomes in the male and female pronucleus at the socially distanced chromosomes (SDC)-positioning stage, when the maternal and paternal chromosome sets reach the maximum distance. **(C)** Table of % SDC positioning during mitosis (n = 11 embryos for WT and *spd-3(oj35)*). **(D)** Representative fluorescence confocal images of WT (left) and *spd-3(oj35)* (right) embryos expressing GFP::β-tubulin, mCherry::γ-tubulin, and mCherry::histone H2B. NEBD indicates the nuclear envelope breakdown stage. SDC indicates the socially distanced chromosome stage observed in *spd-3(oj35)* embryos (right). Scale bar, 10 μm. **(A, D)**, Times are in seconds relative to NEBD (t = 0 s). **(A, B)** Error bars are SD. n = 11 embryos for WT and *spd-3(oj35)*. The significance of differences between results was determined by two-tailed *t* tests (****P < 0.0001).

To characterize the effects on mitotic spindle alignment in *spd-3(oj35)*, we filmed embryos coexpressing mCherry::γ-tubulin and mCherry::histone H2B (Video 2). Following fertilization in control embryos, the pronuclear–centrosomal complex moves to the center and rotates to align the mitotic spindle along the anterior/posterior axis (A/P axis) (Fig S2A). This rotation is impaired in *spd-3(oj35)* embryos, resulting in a mitotic spindle, that is, misaligned to the A/P axis. Consistent with the previous report (Dinkelmann et al, 2007), we observed increased spindle misalignment at anaphase onset in one-cell stage *spd-3(oj35)* embryos (Fig S2B). Moreover, the metaphase spindles in the *spd-3(oj35)* embryos are shorter in comparison to control spindles (Fig S2C, control 14.8 ± 0.6 μm (Stdev), *spd-3(oj35)* 12.7 ± 1.4 μm) and centrosomes are enlarged in the *spd-3(oj35)* (Fig S2D). Quantification of the duration of mitosis in one-cell stage embryos revealed that mitosis is prolonged in the *spd-3(oj35)* mutant embryos. Although the *spd-3(oj35)* mutant embryos display a similar duration of the time from pronuclear meeting to NEBD (Fig S2E), the duration from NEBD to anaphase onset, is extended from 180.0 ± 26.0 s in control embryos to 272.7 ± 60.3 s in *spd-3(oj35)* (Fig 1A). We also observed that although control embryos formed metaphase plates at around 120 s, *spd-3(oj35)* embryos had not formed metaphase plates at this time point. These results suggest that *spd-3(oj35)* does not only affect spindle positioning but also spindle length and the timing of mitotic events.

Video 2 Live-cell imaging of WT and *spd-3(oj35)* embryos during mitosis. The WT (left) and *spd-3(oj35)* (right) embryos coexpressing GFP::β-tubulin, mCherry::γ-tubulin, and mCherry::histone H2B. Fluorescence confocal images were acquired every 15 s by collecting 17 z-planes at 1.0-μm intervals. Sale bar represents 10 μm.Download video

### The SPD-3 is critical for mitotic chromosome positioning during congression

During our analysis of mitosis in the *spd-3(oj35)* embryos we noticed that the mutant embryos display an additional unusual phenotype during chromosome positioning and alignment. Instead of forming a normal metaphase plate in the center of the embryo, the individual sets of chromosomes (paternal and maternal) are initially positioned on the periphery of the respective nuclei, giving rise to a transient diamond–shaped spindle configuration (Fig 1B–D, Video 2), before converging to form a metaphase plate. Inspired by the current pandemic, we called this abnormal positioning stage the “socially distanced chromosome (SDC) positioning” (Fig 1D and Video 1) as the two individual sets of chromosomes, paternal and maternal, showed a large physical distance (Fig 1B). In addition, although control embryos form metaphase spindles at ∼120 s after NEBD, spindles of *spd-3(oj35)* were still in the SDC state at this point. Once a metaphase plate forms in the *spd-3(oj35)* embryos, it is significantly wider (Fig S2F, control: 5.6 ± 0.2 μm, *spd-3(oj35)*: 7.3 ± 1.4 μm) in comparison to control metaphase plates. This observation suggests an additional role for SPD-3 during chromosome congression.

### SPD-3 mutation leads to changes in microtubule dynamics

**Figure S3.**
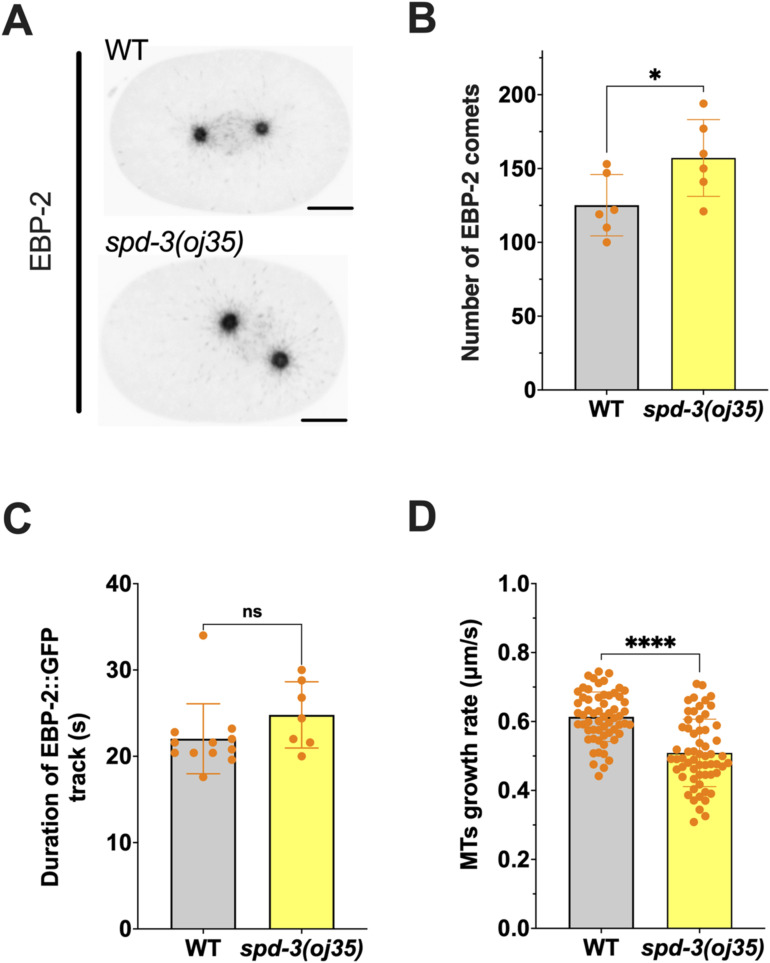
Microtubules (MTs) dynamics are affected by *spd-3(oj35)* mutation, related to Fig 1. **(A)** Spinning-disk confocal images of embryos in WT (top) and *spd-3(oj35)* (right). Embryos are coexpressing mCherry::histone H2B, and EBP-2::GFP. Scale bars, 10 μm. **(B)** Plot of the number of EBP-2 comets per centrosome in WT (gray bar, n = 6) and *spd-3(oj35)* embryos (yellow bar, n = 6). **(C)** Duration of EBP-2::GFP track in WT and *spd-3(oj35)* embryos. **(D)** Plot of MT growth rate in WT and *spd-3(oj35)* embryos. **(C, D)** Each dot represents the selected MT labeled by EBP-2::GFP in embryo. **(B, C, D)** Error bar are SD. The significance of differences between results was determined by two-tailed *t* tests (ns, not significant difference; *P < 0.05; ****P < 0.0001).

Previous publications have shown that changes in microtubule nucleation and growth rate could affect spindle positioning. As an example, RNAi of ZYG-9 in *C. elegans* leads to pronuclear migration defects, and the spindle forms at the posterior, orients incorrectly, and contains unusually short microtubules. It was shown that *zyg-9 (RNAi)* also leads to a strong decrease in microtubule nucleation rate and a nearly threefold decrease in microtubule growth rate (Srayko et al, 2005), which might induce the spindle-positioning phenotype upon ZYG-9 depletion. To determine if changes in microtubule dynamics occurred in *spd-3(oj35)* embryos that could affect spindle and maybe chromosome positioning, we generated a *spd-3(oj35)* strain that expressed EBP-2, a marker of growing microtubule plus-ends (Fig S3A). We quantified microtubule nucleation and growth rate, and the time over which we could track individual EBP-2 comets (Fig S3, Video 3). We found that the number of EBP-2 comets is elevated in *spd-3(oj35)* (157 ± 26) in comparison to control (125 ± 21) (Fig S3B), suggesting an increase in the number of polymerizing microtubules. In addition, microtubule growth rate decreases in *spd-3(oj35)* (0.51 ± 0.1 μm/s) in comparison to control (0.61 ± 0.01 μm/s) (Fig S3D). The duration of individual tracks is not significantly different (22 ± 4.1 s in control and 24.8 ± 3.8 s in *spd-3(oj35)*) (Fig S3C). Overall, although there are slightly more growing plus-ends detected in *spd-3(oj35)* that grow with a decreased velocity, the approximate total length of microtubules, calculated by growth rate and duration of track, is only marginally different. In addition, the change in microtubule growth rate in the *spd-3(oj35)* is rather small, that is, in comparison to the ∼threefold reduction in ZYG-9–depleted embryos, suggesting that this might not be the main driver of spindle misalignment.

Video 3 Live-cell imaging of WT and *spd-3(oj35)* expressing EBP-2::GFP embryos during mitosis. The WT (left) and *spd-3(oj35)* (right) embryos expressed EBP-2::GFP. Fluorescence confocal images were acquired at 400 m sec intervals for 1 min. Sale bar represents 10 μm.Download video

### The SAC does not contribute to the delay in anaphase onset but reduces chromosome and spindle alignment defects in *spd-3(oj35)*

**Figure 2.**
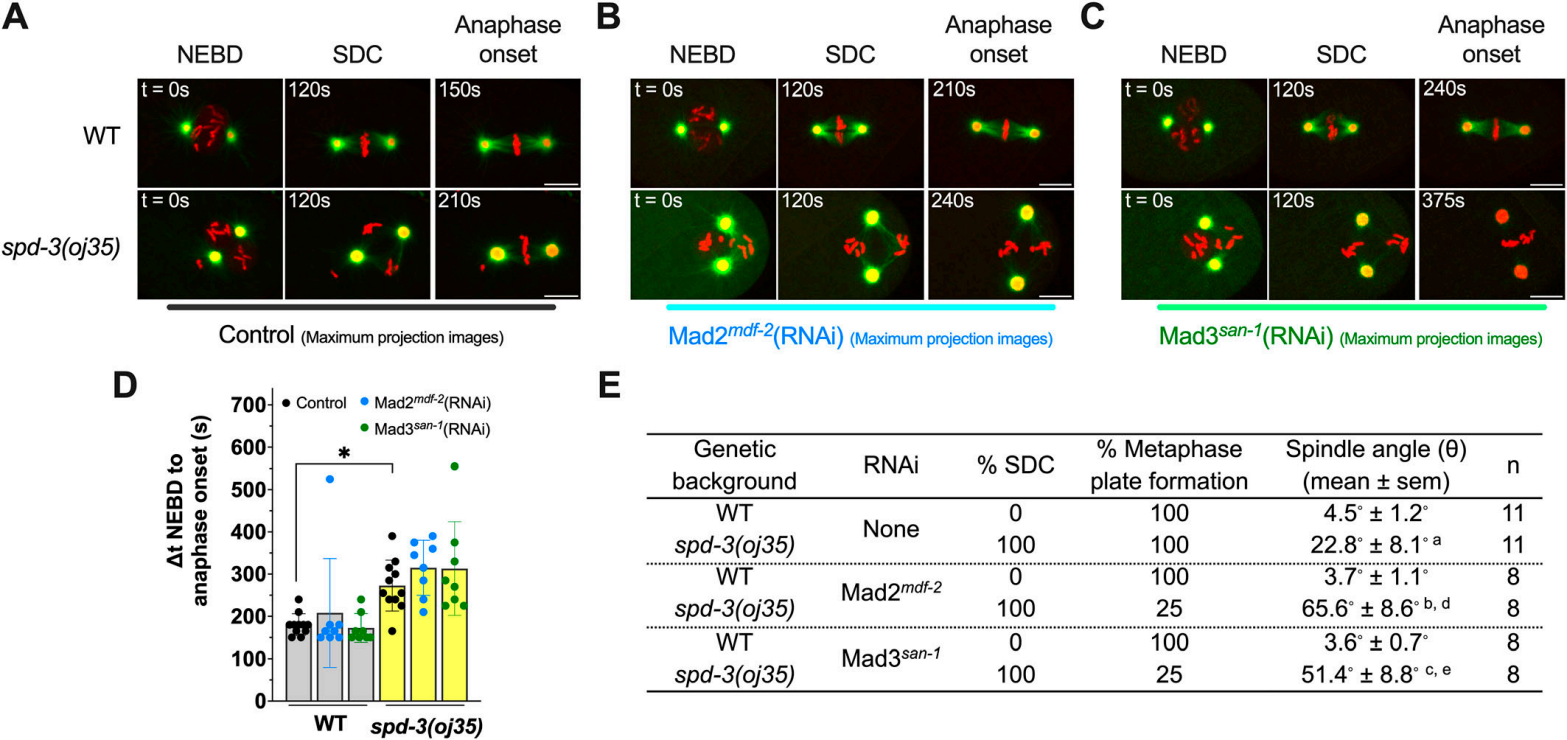
Spindle assembly checkpoint affected by *spd-3(oj35)* mutation. **(A, B, C)** Representative fluorescence confocal images of WT and *spd-3(oj35)* embryos coexpressing GFP::β-tubulin, mCherry::γ-tubulin, and mCherry::histone H2B treated without ((A), n = 11), with Mad2^*mdf-2*^ ((B), n = 8), or with Mad3^*san-1*^ ((C), n = 8) RNAi. Scale bar, 10 μm. Times are in seconds relative to NEBD (t = 0 s). **(D)** Graph plotting the duration between NEBD and chromosomes segregation in WT (gray bar) and *spd-3(oj35)* (yellow bar) treated without (black dots), and with Mad2^*mdf-2*^*(RNAi)* (blue dots) or with Mad3^*san-1*^*(RNAi)* (green dots). **(E)** Table of mitotic events scored in control-, Mad2^*mdf-2*^-, and Mad3^*san-1*^-depleted embryos. Significance is indicated by a, b, c, d, and e: “a” indicates *P* < 0.05 in comparison to WT. “b” indicates *P* < 0.01 in comparison to *spd-3(oj35)*. “c” indicates *P* < 0.01 in comparison to *spd-3(oj35)*. “d” indicates *P* < 0.0001 in comparison to WT treated with *mdf-2 (RNAi)*. The “e” indicates *P* < 0.001 in comparison to WT treated with *san-1* (RNAi). Error bars are SD. One-way ANOVA was used for statistical analysis (****P < 0.0001).

Our analysis of the *spd-3(oj35)* showed an increase in the duration from NEBD to anaphase onset, suggesting a potential delay in anaphase onset. As anaphase onset is regulated by the SAC (Lischetti & Nilsson, 2015; O’Connor et al, 2015), we tested if the delay in mitotic timing is due to activation of the SAC by depleting the checkpoint proteins MDF-2/Mad2 and SAN-1/Mad3 in *spd-3(oj35)* embryos (Fig 2A–C, Video 4). To quantify the effect of SAC, we measured the timing between NEBD and anaphase onset in control embryos and *spd-3(oj35)* with and without Mad2 and Mad3 (Fig 2E). This analysis did not show any changes in the duration from NEBD to anaphase onset with or without those SAC proteins. In any condition, the duration was still prolonged in *spd-3(oj35)* after depletion of Mad2 and Mad3. However, we noticed that in *spd-3(oj35)* embryos, the treatment of *mdf-2*/Mad2(RNAi) and *san-1*/Mad3(RNAi) often resulted in chromosome segregation that occurred before or in absence of the formation of a metaphase plate (six out of eight embryos), suggesting defects in chromosome congression or alignment, and an increase in spindle alignment defects (eight out of eight embryos) (Fig 2B–D). Overall, the data indicate that the SAC is not responsible for the delay in anaphase onset but is contributing to reducing the chromosome and spindle alignment defects in the *spd-3(oj35)*.

Video 4 Live-cell imaging of Mad2^*mdf-2*^- and Mad3^*san-1*^-depleted embryos during mitosis. The WT (top) and *spd-3(oj35)* (bottom) embryos coexpressing GFP::β-tubulin, mCherry::γ-tubulin, and mCherry::histone H2B without (left), and with Mad-2^*mdf-2*^*(RNAi)* (middle) or Mad3^*san-1*^*(RNAi)* treatment. Fluorescence confocal images were acquired every 15 s by collecting 17 z-planes at 1.0-μm intervals. The images were processed by maximum projection. Sale bar represents 10 μm.Download video

### The mitotic phenotypes of the *spd-3(oj35)* mutant are not caused by changes in energy metabolism

**Figure S4.**
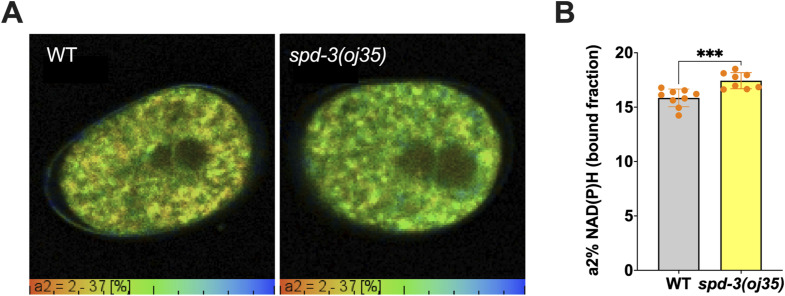
FLIM NAD(P)H imaging shows increased metabolic rates in *spd-3(oj35)* embryos, related to Fig 1. **(A)** Images showing color-coded values of NAD(P)H bound fraction in WT and *spd-3(oj35)* one-cell stage *C. elegans* embryo. Bottom gradient indicates the level of bound NAD(P)H reaching from 2–37%. **(B)** Bar chart graph comparing mean values for NAD(P)H bound fraction (a2%) in tested embryos. Mean pixel value was calculated from two to three image acquisitions per embryo. n = 9 for WT and n = 8 for *spd-3(oj35)*. Error bars are SD. The significance of differences between results was determined by two-tailed *t* tests (***P < 0.001).

SPD-3 was identified as a mitochondria-localizing protein based on colocalization of SPD-3::GFP and mitochondria stained with MitoTracker Red CMXRos (MTR) (Fig S1B) and immuno-EM (Dinkelmann et al, 2007; Labrador et al, 2013). We wanted to determine the mechanisms of how the mitochondrial protein SPD-3 affects spindle and chromosome positioning and anaphase onset. As it was previously suggested that defects in the mitochondrial function in the *spd-3(oj35)* could lead to reduced function of cytoskeletal motors, possibly caused by variations in ATP levels, we measured the metabolic rates in *spd-3(oj35)* embryos by fluorescence lifetime imaging microscopy (FLIM) of NADH autofluorescence (Schneckenburger, 1992; Sud et al, 2006). FLIM NADH offers a marker-free readout of the mitochondrial function of cells in their natural microenvironment and allows different pools of NADH to be distinguished within a cell (Blacker et al, 2014). NADH autofluorescence can be used as a marker of cellular redox state and indirectly also of cellular energy metabolism (Schneckenburger, 1992; Sud et al, 2006). These measurements show that metabolic rates in *spd-3(oj35)* embryos are increased in comparison to WT (Fig S4A and B). This agrees with previously reported increased ATP levels (Dinkelmann et al, 2007).

To test if elevated (or reduced) ATP could lead to similar mitotic phenotypes, we examined the effect of depleting proteins that are part of the electron transfer chain (ETC) in *C. elegans* (Dancy, 2015) (Table S1). CLK-1 is required for the biosynthesis of ubiquinone, a carrier in the ETC, and depletion of CLK-1 leads to elevated ATP levels (Braeckman et al, 1999; Dancy, 2015). We screened embryos of the null allele *clk-1(qm30)* for defects in spindle and chromosome positioning but could not reproduce any of the observed phenotypes of the *spd-3(oj35)* mutant in respect to spindle and chromosome positioning. We further analyzed the effects of *isp-1(qm150)*, *ucr-1 (RNAi)*, *mev-1 (RNAi)*, or *cco-1 (RNAi)*, which alter the ETC function, resulting in decreased ATP levels (Dillin et al, 2002; Dancy, 2015). Although we did observe spindle misalignment phenotypes in all examined depletions, we never detected the SDC-positioning phenotype in those mitochondrial mutants and the timing between NEBD and anaphase onset was not affected (Table S1).

Table S1. Strains and RNAi-feeding clones for the electron transfer chain pathway in mitochondria, related to Fig 1.

In summary, although the disruption of some mitochondrial genes did affect spindle alignment, it did not mimic the SDC phenotype, suggesting that metabolic perturbations in *spd-3(oj35)* worms may not directly lead to the observed chromosome-positioning defects. In agreement with this, also treatment of control embryos with mitochondrial inhibitors (Dinkelmann et al, 2007) did not reproduce the SDC *spd-3(oj35)* phenotype, suggesting that the observed phenotype is not based on misregulation of ATP levels alone.

### SPD-3 is important for ER morphology

**Figure 3.**
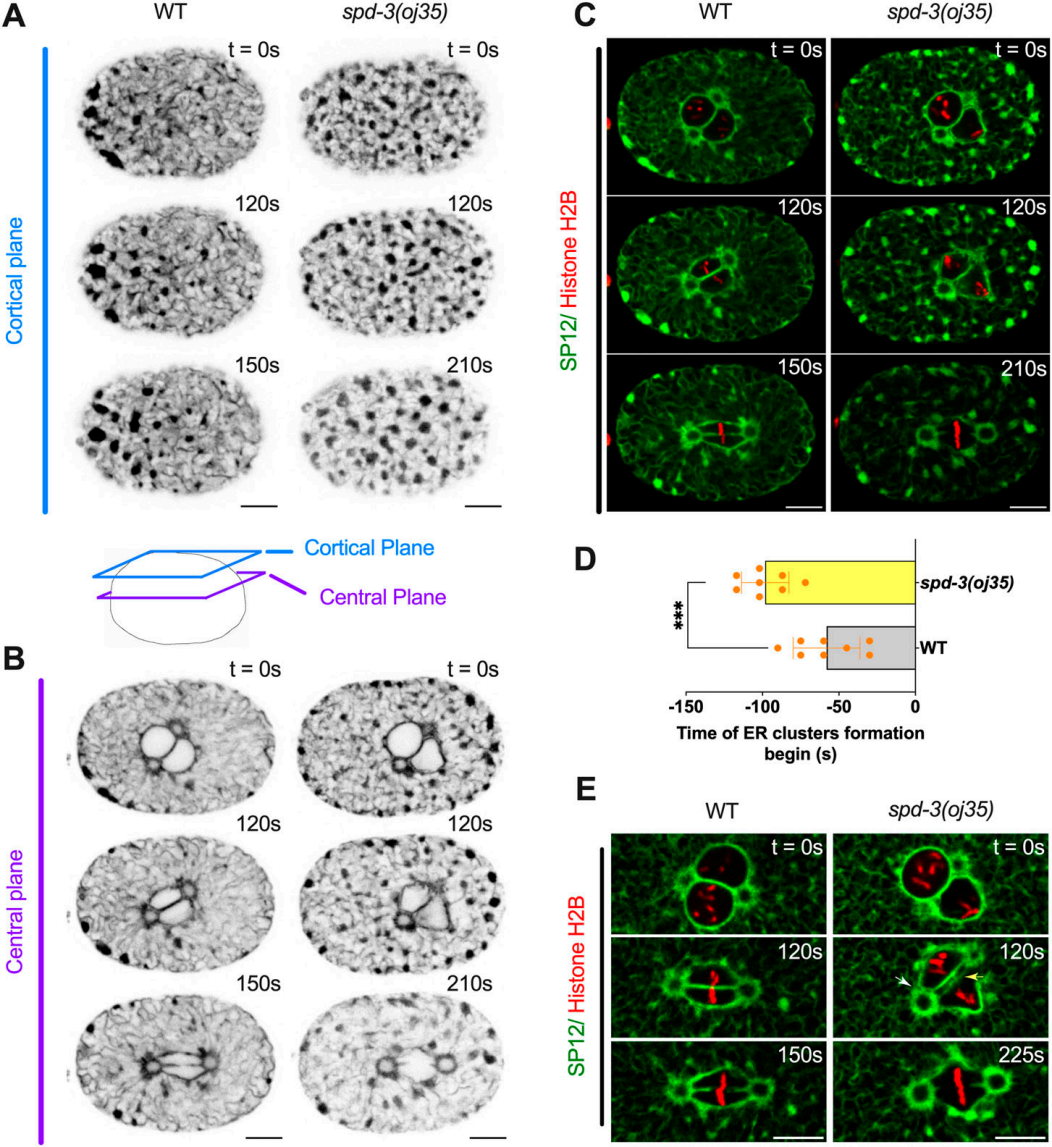
*spd*-3(oj35) embryos show abnormal ER morphology. **(A, B)** Spinning-disk confocal images of WT (left) and *spd-3(oj35)* (right) embryos expressing the ER marker GFP::SP12 and mCherry::histone H2B. **(A, B)** Cartoon indicates the position of a cortical plane just beneath the embryo surface (blue, (A)) and a central plane (purple, (B)) from WT and *spd-3(oj35)* are shown. **(A, B, C)** Merge of the images shown in (A, B). WT is on left and *spd-3(oj35)* is on right. **(D)** Graph plotting the timing of ER cluster formation in the cortical section of embryos expressing GFP::SP12 in mitosis (n = 8 embryos for WT and *spd-3(oj35)*). Error bars are SD. The significance of differences between results was determined by two-tailed *t* tests (***P < 0.001). **(E)** Spinning-disk confocal images of the ER surrounding the pronucleus in WT (left) and *spd-3(oj35)* (right) embryos coexpressing mCherry::histone H2B and GFP::SP12. The white arrow indicates a gap appearing between the centrosome and pronuclei (t = 120 s). The yellow arrow indicates a gap appearing between the pronuclei (t = 120 s). **(A, B, C, D, E)** Times are in seconds relative to NEBD (t = 0 s). Scale bars, 10 μm.

As we observed an effect on the timing from NEBD to anaphase, we decided to further investigate the arrangement of ER and nucleus. The ER surrounds and encloses the pronucleus, forming a characteristic bi-membraned nuclear envelope that protects the chromatin. To determine the arrangement of the ER in the *spd-3(oj35)* mutant, we generated a mutant strain that co-expressed GFP-labeled SP12, an ER lumen marker, and mCherry-labeled histone. Analysis of this strain revealed that the morphology of the ER is severely affected in the *spd-3(oj35)* mutant (Fig 3A–C and Video 5). The ER shows an increase in the formation of ER clusters and drastic changes in the morphology surrounding the nucleus (Fig 3B and C). In general, the distribution of ER clusters is asymmetric (Poteryaev et al, 2005), with more clusters localizing in the anterior half of the embryo in the vicinity of the cortex (Fig 3A). In control embryos, clusters begin to form 58.1 ± 21.9 s before NEBD (Fig 3D) and become enriched around the centrosome and mitotic spindle (Fig 3B and C). In the *spd-3(oj35)* mutant, ER clusters begin to form earlier, at 98.3 ± 15.5 s before NEBD (Fig 3D) and are found in the cytoplasm, around the nucleus and on the cortex in both the anterior and posterior sides of the *spd-3(oj35)* embryo (Fig 3A–C). In addition, gaps appear in between centrosome and pronuclei (white arrow) and in between pronuclei (yellow arrow) before anaphase onset in *spd-3(oj35)* embryos (Fig 3E). These results show that ER morphology is affected in the early embryonic stage of the s*pd-3(oj35)*, suggesting a potential role for SPD-3 in these processes.

Video 5 Live-cell imaging of WT and *spd-3(oj35)* expressing GFP::SP12 embryos during mitosis. The WT (left) and *spd-3(oj35)* (right) embryos coexpressing GFP::SP12 and mCherry::histone H2B. Fluorescence confocal images were acquired every 15 s by collecting 17 z-planes at 1.0-μm intervals. Sale bar represents 10 μm.Download video

**Figure S5.**
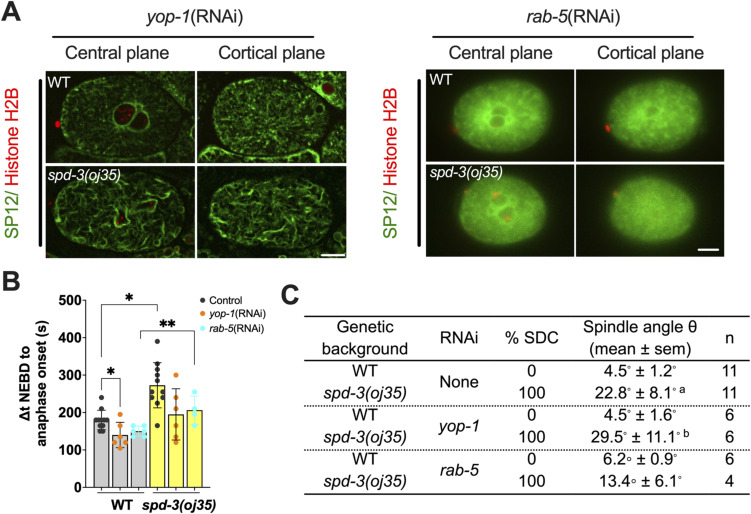
Socially distanced chromosomes positioning is not rescued by the removal of ER clusters, related to Fig 3. **(A)** Left: spinning-disk confocal images of embryos in WT (top) and *spd-3(oj35)* (bottom) with *yop-1(RNAi)*. Right: wide-field microscopy images of embryos in WT (top) and *spd-3(oj35)* (bottom) with *rab-5(RNAi)*. Representative images of a central (left) and cortical plane (right) are shown. Embryos express the GFP::SP12 and mCherry::histone H2B. Scale bar, 10 μm. **(B)** Graph plotting prometaphase duration as defined by the time between NEBD and anaphase onset in WT (gray bar) and *spd-3(oj35)* (yellow bar) treated without (black dots) or with *yop-1(RNAi)* (orange dots) or *rab-5(RNA)* (turquoise dots). Error bars are SD. The significance of differences between results was determined by two-tailed *t* tests (*P < 0.05). **(C)** Table of the percentage of socially distanced chromosomes phenotype and angle of spindle orientation in WT and *spd-3(oj35)* embryos treated without or with *yop-1* (RNAi) or *rab-5* (RNAi). The “a” indicates *P < 0.05 in comparison to WT. The “b” indicates *P < 0.05 in comparison to WT treated with *yop-1* (RNAi).

To determine the potential effects of ER morphology on spindle and chromosome positioning, we analyzed a number of ER mutants that show drastic changes in ER morphology, in particular, the ratio of clusters to tubes. The RAB-5 and YOP-1/RET-1 proteins are important for regulating the ER morphology during mitosis and controlling the kinetics of nuclear envelope disassembly (Audhya et al, 2007). The endosomal Rab-type GTPase, RAB-5, plays a role in the homotypic fusion of ER membranes (Audhya et al, 2007). The YOP-1, homologs of DP1/NogoA, localizes to the ER and plays a functionally redundant role in generating tubular morphology in the ER (Audhya et al, 2007). In YOP-1– and RAB-5–depleted embryos, there are fewer thick ER tubules that are poorly organized and no mitotic ER clusters are formed. We treated *spd-3(oj35)* worms with *rab-5 (RNAi)* and *yop-1 (RNAi)* with the aim to reduce the number of ER clusters in the cytoplasm. Although we did observe fewer and smaller ER clusters in the *spd-3(oj35)* mutant after *rab-5* and *yop-1* depletion (Fig S5A), the SDC phenotype was still present (Fig S5C). We also did not observe a decrease in the spindle orientation defect after *yop-1 (RNAi)*; however, after *rab-5 (RNAi)*, the spindle misalignment was slightly reduced from 22.8 ± 8.1 in *spd-3(oj35)* to 13.4 ± 6.1 in *spd-3(oj35)* treated with *rab-5 (RNAi)* (Fig S5C). Interestingly, *yop-1(RNAi)* and *rab-5 (RNAi)* lead to a decrease in the duration between NEDB and anaphase onset (Fig S5B). In *C. elegans*, it was shown that depletion of RAB-5 and co-depletion of YOP-1 and RET-1 leads to changes in the ER structure and a delay of nuclear envelope disassembly (Audhya et al, 2007). As we quantified the time between NEBD and anaphase onset, it is possible that a delay in NEBD upon *rab-5* and *yop-1(RNAi)* could shorten the overall duration of this time period in *spd-3(oj35)*. It is however also possible that the extended duration in *spd-3(oj35)* is a consequence of disrupting the ER structure. In summary, this data suggest that the SDC phenotype observed in the *spd-3(oj35)* is unrelated to defects in the ER structure, whereas the duration from NEBD to anaphase onset and the spindle mis-orientation could be in part induced by the ER cluster formation.

### Structural changes in the ER could be induced by blocking UPR^ER^ but do not affect chromosome positioning

**Figure S6.**
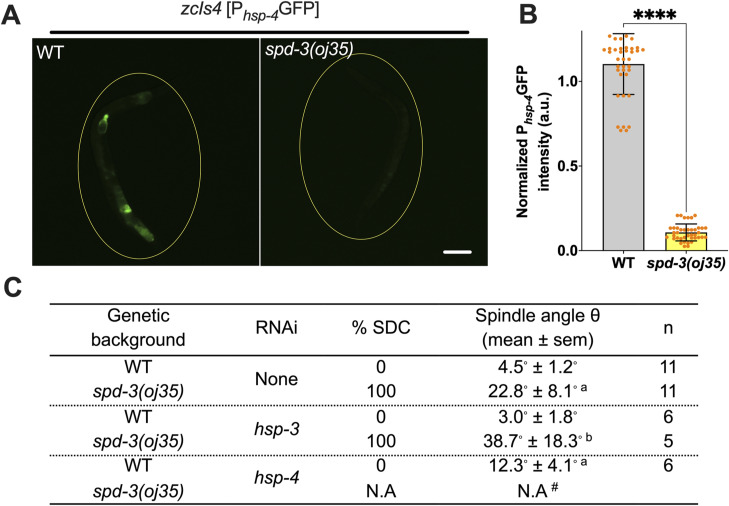
Reduction of *hsp-4::gfp* in *spd-3(oj35)* adult animals, related to Fig 3. **(A)** Fluorescence images of WT and *spd-3(oj35)* adult animals carrying the P_*hsp-4*_∷GFP (*zcIs4*) transgene. Scale bar, 100 μm. **(B)** Graph plotting the normalized overall fluorescence of *zcIs4* in WT (n = 40 worms) and *spd-3(oj35)* (n = 40 worms). Error bars are SD. The significance of differences between results was determined by two-tailed *t* tests (****P < 0.0001). **(C)** Table of the percentage of socially distanced chromosomes phenotype and spindle orientation angle in WT and *spd-3(oj35)* embryos treated without or with *hsp-3* (RNAi) or *hsp-4* (RNAi). The “a” indicates *P < 0.05 in comparison to WT. The “b” indicates *P < 0.05 in comparison to *spd-3(oj35)* treated with *hsp-3* (RNAi). The “#” symbol indicates the complete absence of analyzable embryos in the one-cell stage among the 100 screened embryos.

Next, as ER dynamics and morphology changes can also be induced by ER stress, we tested whether the ER stress–unfolded protein response (UPR^ER^) is changed in the *spd-3(oj35)* mutant. During *C. elegans* UPR^ER^, two homologs of the ER-resident heat-shock protein BiP, HSP-3, and HSP-4 are essential for the formation of sheet-like ER structures (Poteryaev et al, 2005). Depletion of HSP-3 leads to up-regulation of the *hsp-4* gene in *C. elegans* (Kapulkin et al, 2005). To examine the UPR^ER^ in *spd-3(oj35)*, we imaged worms expressing the *zcIs4* transgene, a transcriptional fusion of the *hsp-4* promoter to GFP that can be used to quantify HSP-4 expression levels (Calfon et al, 2002). The *zcIs4* reporter is strongly up-regulated in response to treatments inducing ER stress and can thus function as a readout for UPR^ER^ activity (Calfon et al, 2002). We found an overall lower HSP-4 expression level in *spd-3(oj35)* mutant worms by quantification of fluorescence levels, indicating an approximately 11-fold decrease in *hsp-4::gfp* expression (Fig S6A and B). This result suggested that changes in ER morphology could be induced by blocking UPR^ER^, leading to low HSP-4 expression levels in the *spd-3(oj35)* mutant. To further determine whether the mitotic defects could be rescued by increasing HSP-4, we treated *spd-3(oj35)* mutant with *hsp-3 (RNAi)* to increase the endogenous *hsp-4* transcription (Kapulkin et al, 2005). However, the spindle and chromosome-positioning defects in the *spd-3(oj35)* mutant are not prevented by *hsp-3 (RNAi)* (Fig S6C). Because there was no strong effect on spindle and chromosome positioning and metaphase progression we did not quantify the duration between NEBD and anaphase onset. Previous publications showed that *hsp-3 (RNAi)* does not affect ER dynamics in the early embryo. In contrast, when HSP-4 function is reduced by RNAi, the ER accumulates in multiple foci, and the ER morphology is distinctly disrupted (Poteryaev et al, 2005). We did not observe any effects on chromosome positioning and spindle defects in HSP-3–depleted embryos (Fig S6C). In control embryos treated with *hsp-4 (RNAi)*, we saw a slight increase in spindle misalignment, from 4.5 ± 1.2 in untreated to 12.3 ± 4.1 in *hsp-4 (RNAi)*–treated embryos (Fig S6C). Depletion of HSP-4 in *spd-3(oj35)* resulted in severely affected embryos that could not be analyzed, suggesting that *hsp-4 (RNAi)* reduced the viability of *spd-3(oj35)*. Taken together, our results suggest that perturbation of the mitochondrial protein SPD-3 affects the morphology of the ER and that this might be induced by the UPR^ER^ regulatory system. However, the structural changes of the ER are most likely not responsible for the observed chromosome positioning defects but could contribute to the spindle alignment defects in the *spd-3(oj35)* mutant embryos.

### SPD-3 is critical for nuclear envelope structure and dynamics

**Figure S7.**
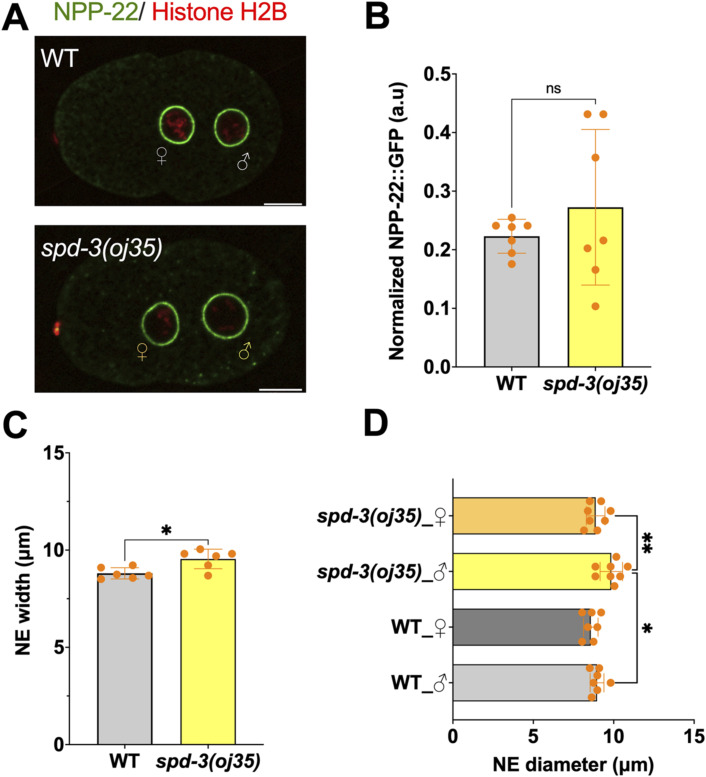
The male pronucleus expands before the pronuclear meeting, related to Fig 4. **(A)** Representative images of embryos coexpressing NPP-22::mNG and mCherry::histone H2B in WT and *spd-3(oj35)* embryos. Scale bars, 10 μm. **(B)** Plot the intensity of NPP-22::mNG in the nuclear membrane of WT (n = 7) and *spd-3(oj35)* (n = 8). **(C)** Plot of the pronuclear envelope width in WT and *spd-3(oj35)*. **(D)** Plot the nuclear envelope width of male and female pronuclei in WT and *spd-3(oj35)*. **(B, C, D)** Error bars are SD. The significance of the difference between strains was determined by *t* tests (ns indicates not significant difference. *P < 0.05. **P < 0.01).

**Figure 4.**
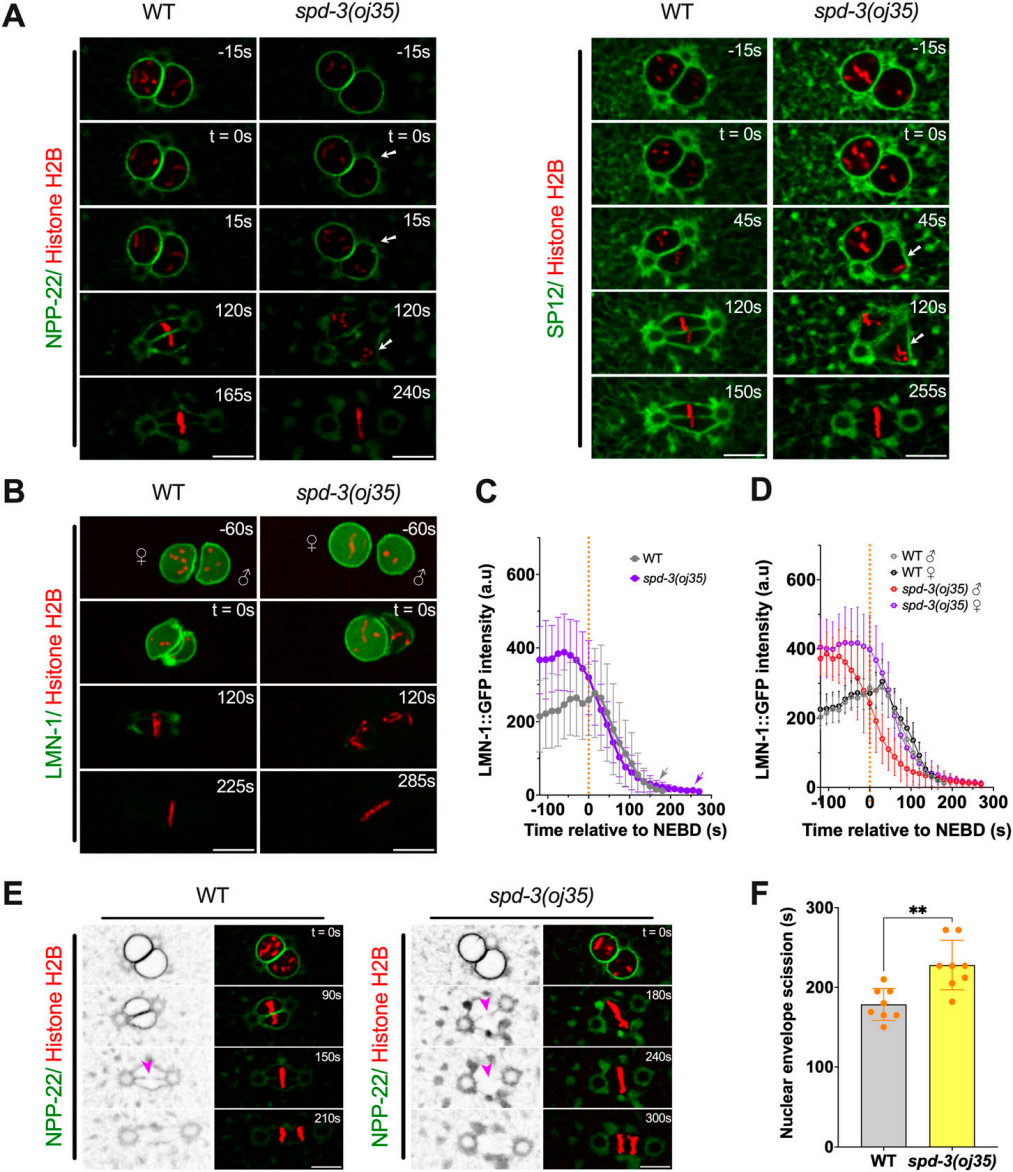
SPD-3 is involved in structural integrity and disassembly of the nuclear envelope during mitosis. **(A)** Spinning-disk confocal images of nuclear morphology in WT and *spd-3(oj35)* embryos coexpressing mCherry::histone H2B and NPP-22::mNG (left) or GFP::SP12 (right). The appearance of a deformed NE shape around NEBD in mitosis is indicated by the white arrow. **(B)** Time-lapse sequences of WT (left) and *spd-3(oj35)* (right) embryos expressing GFP::LMN-1 and mCherry::histone H2B. ♀ indicates female pronucleus and ♂ indicates male pronucleus. **(C, D)** The total fluorescence intensity of LMN-1 was measured at each time point between 120 s before NEBD to anaphase onset for WT (gray line, n = 7) and *spd-3(oj35)* (purple line, n = 11). The mean value for this measurement is plotted versus time. Orange dashed line represents NEBD as t = 0 s. Gray arrow indicates the anaphase onset in WT and purple arrowhead indicates the anaphase onset in *spd-3(oj35)*. **(D)** Plot of the timing of nuclear lamin disassembly in the female (black line/purple line) and male pronucleus (gray line/red line) in WT and *spd-3(oj35)* embryos. **(E)** Fluorescence confocal images of control and *spd-3(oj35)* embryos expressing NPP-22::mNG and mCherry::histone H2B. Magenta arrowheads mark the site of NE scission in WT (left) and *spd-3(oj35)* (right). **(F)** Graph plotting time of NE scission in WT (n = 8) and *spd-3(oj35)* (n = 8) embryos. **(A, B, C, D, E, F)** Times are in seconds relative to NEBD (t = 0 s). Scale bars, 10 μm. **(C, D, F)** Error bars are SD. The significance of the difference between results was determined by two-tailed *t* tests (**P < 0.01).

Our analysis of the ER morphology revealed a change in the shape of the pronucleus in *spd-3(oj35)* mutants. As the ER and the nucleus are intimately connected, we generated strains that carry either NPP-22::mNeonGreen (NPP-22::mNG) (Mauro et al, 2022) (Video 6), a conserved transmembrane nucleoporin, or GFP tagged lamin (Link et al, 2018), which enabled us to analyze the shape and organization of the nuclear envelope during mitosis. Analysis of these strains by light-microscopy showed that the nuclear shape is altered, beginning at around NEBD, in *spd-3(oj35)*, consistent with the observed changes in ER morphology surrounding the spindle (Figs S7 and 3A). As previously described, we also found that NPP-22 localizes not only to the nuclear envelope but also accumulates in the ER around the centrosomes, the centriculum (Maheshwari et al, 2023; Nkoula et al, 2023). In addition, we observed NPP-22 also localizing to larger structures around the nucleus, which most likely reflect a localization to ER (Fig 4A). Analysis of the LMN-1::GFP in the *spd-3(oj35)* mutant background showed that nuclear lamin is disassembled completely around 180 s after NEBD in both WT and *spd-3(oj35)* embryos (Fig 4B–D and Video 7). However, the lamin of the paternal pronucleus disappears earlier than the lamin on the maternal pronucleus in *spd-3(oj35)* embryos. This asymmetry in nuclear disassembly is not observed in control embryos (Fig 4D), suggesting that the structural integrity of the nucleus could be impaired in the *spd-3(oj35)* mutant.

Video 6 Live-cell imaging of WT and *spd-3(oj35)* expressing NPP-22::GFP embryos during mitosis. The WT (left) and *spd-3(oj35)* (right) embryos coexpressing NPP-22::GFP and mCherry::histone H2B. Fluorescence confocal images were acquired every 15 s by collecting 17 z-planes at 1.0-μm intervals. Sale bar represents 10 μm.Download video

Video 7 Live-cell imaging of WT and *spd-3(oj35)* expressing LMN-1::GFP embryos during mitosis. The WT (left) and *spd-3(oj35)* (right) embryos coexpressing LMN-1::GFP and mCherry::histone H2B. Fluorescence confocal images were acquired every 15 s by collecting 17 z-planes at 1.0-μm intervals. Sale bar represents 10 μm.Download video

**Figure S8.**
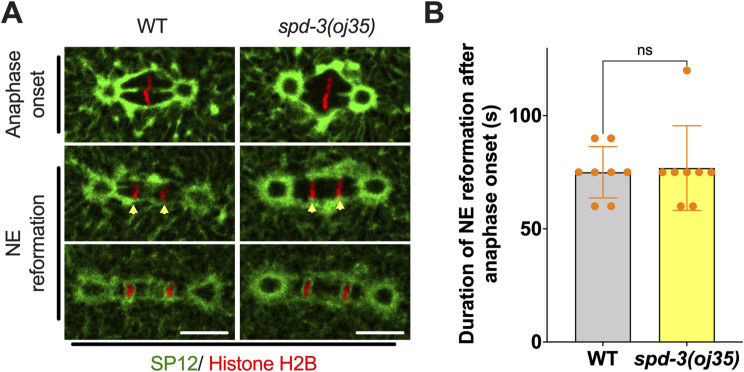
Duration of NE assembly in WT and *spd-3(oj35)* embryos, related to Fig 4. **(A)** Fluorescence confocal images of WT and *spd-3(oj35)* embryos expressing GFP::SP12 and mCherry::histone H2B. Yellow arrowheads mark the site of NE reformation in WT (left) and *spd-3(oj35)* (right). scale bars, 10 μm. **(B)** Graph plotting duration of NE reformation after anaphase onset in WT (left, n = 8) and *spd-3(oj35)* (right, n = 8). Error bars are SD. The significance of the difference between strains was determined by two-tailed *t* tests (ns, not significant difference).

To examine NE assembly, we checked the timing and duration of NE reformation in anaphase (Fig S8A). In WT embryos, the reformation of new NE around the segregated chromosomes begins approximately 86.7 ± 20.9 s after anaphase onset and the new NE is completed after 161.7 ± 27.8 s. The reassembly of the NE is slightly delayed in *spd-3(oj35)* embryos as it is initiated 101.7 ± 18.0 s and completed 182.0 ± 29.0 s after anaphase onset. Although NE reformation around the segregated chromosomes is a little bit delayed in the *spd-3(oj35)* embryos, the duration of NE reformation does not differ between control and *spd-3(oj35)* embryos (Fig S7B).

The timely disassembly of the nuclear lamina is required for chromosome alignment and NE scission to allow parental chromosomes to merge on the metaphase plate in *C. elegans* (Chase et al, 2000; Rahman et al, 2015; Velez-Aguilera et al, 2020). We next examined whether NE scission changes between the juxtaposed pronuclei by labeling of NE (NPP-22::mNG). The NE scission event occurs 178.6 ± 20.0 s after NEBD, or less than 3 s before anaphase onset in control embryos, when the formation of a membrane gap is visible (Fig 4E and F). In contrast, the NE scission event occurs 228.0 ± 31.1 s after NEBD, but ∼50 s before anaphase onset, in *spd-3(oj35)* embryos (Fig 4E and F). Combined these results suggest that SPD-3 is critical for the dynamics of NE disassembly during the first mitotic division in *C. elegans*.

### *spd-3(oj35)* mutants show PLK-1 overexpression

**Figure 5.**
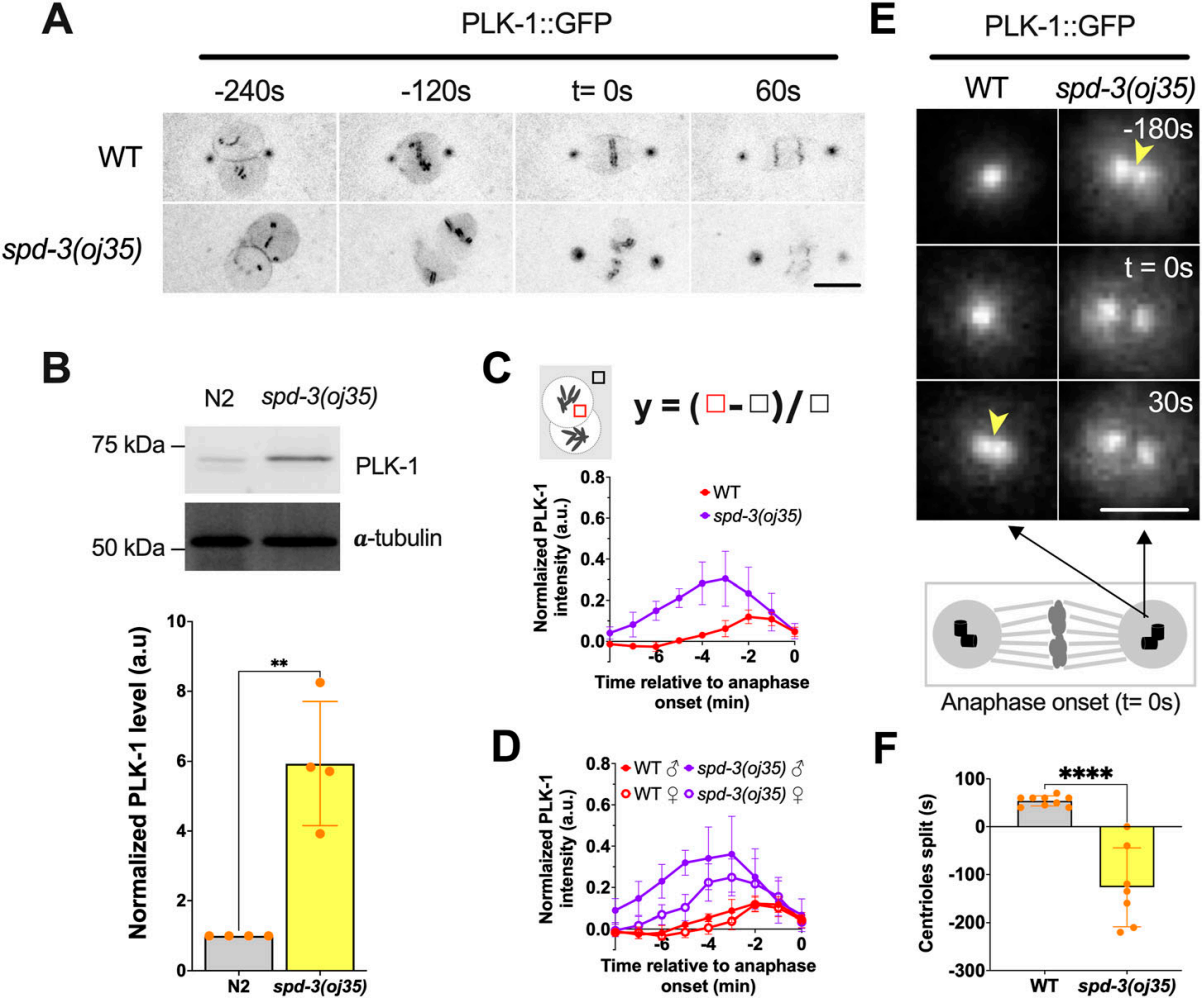
PLK-1 overexpression in the *spd-3(oj35)* mutant. **(A)** Time-lapse sequences of embryos expressing PLK-1::GFP in WT (top) and *spd-3(oj35)* (bottom). Time point 0 is anaphase onset. Scale bar, 10 μm. **(B)** Top: Western blot of PLK-1 protein in N2 (n = 4) and *spd-3(oj35)* (n = 4) worms, bottom: quantification of PLK-1 expression level. **(A, C, D)** Quantification of normalized PLK-1::GFP intensity in the nucleoplasm of embryos measured from time-lapse sequences as in (A). **(C)** The value of PLK-1::GFP intensity in WT (red, n = 6) and *spd-3(oj35)* (purple, n = 6). **(D)** The value of PLK-1::GFP intensity in the nucleoplasm of male and female pronuclei. Solid circles indicate the male pronucleus (n = 6), empty circles indicate the female pronucleus (n = 6). Cartoon showing the method used for quantification of the PLK-1::GFP fluorescence in nucleoplasm. Briefly a fixed-size box is drawn in nucleoplasm (red box) and in cytoplasm (black box) as background at each time point. The normalized PLK-1 level is calculated as ([integrated intensity in red box—integrated intensity in black box]/integrated intensity in black box). ♀ indicates female pronucleus and ♂ indicates male pronucleus. **(E)** Spinning-disk confocal images of the centrosome in WT (left) and *spd-3(oj35)* (right) embryos expressing PLK-1::GFP. Yellow arrowheads indicate the time of centriole splitting. Schematic showing that each centrosome contains a pair of centrioles. Black arrows indicate a centrosome. Scale bar, 2 μm. **(F)** Graph plotting the time of detectable centriole pair splitting in control (left, n = 6 centrosomes) and *spd-3(oj35)* (right, n = 6 centrosomes). **(A, C, D, E, F)** Times are in seconds relative to anaphase onset (t = 0 s). **(B, C, D, F)** Error bars are SD. The significance of the difference between strains was determined by two-tailed *t* tests (**P < 0.01; ****P < 0.0001).

**Figure S9.**
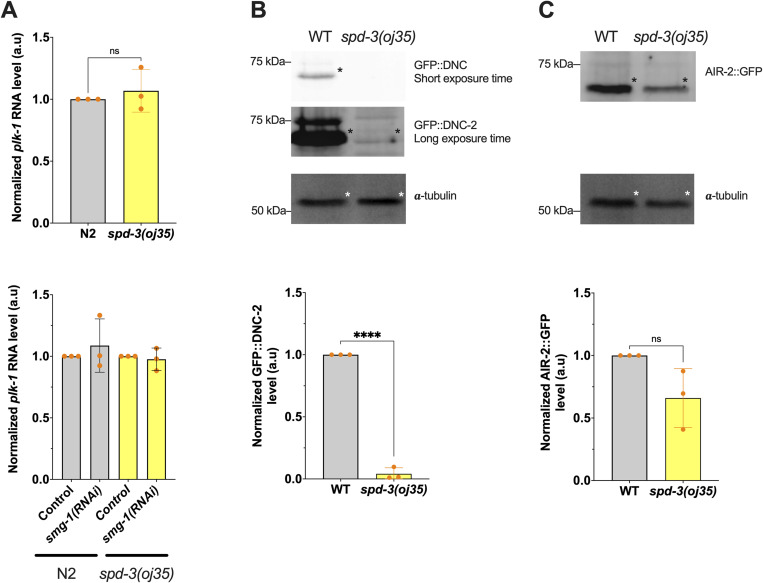
Reduction of GFP::DNC-2 protein level in *spd-3(oj35)*, related to Fig 5. **(A)** Quantification of *plk-1* RNA levels was performed using RT-qPCR. Total RNA was extracted from N2 (gray bar) and *spd-3(oj35)* (yellow bar) whole worm lysates without (top) or with (bottom) *smg-1(RNAi)*. RT-qPCR was used to detect the expression levels of *plk-1* and *tba-1*. The *plk-1* level was then normalized to the *tba-1* level. **(B)** Western blot analysis and quantification of GFP::DNC-2 expression in the WT and *spd-3(oj35)*. **(C)** Western blot analysis and quantification of AIR-2::GFP expression in the WT and *spd-3(oj35)*. **(B, C)** Immunoblotting was performed on whole worm lysates using anti-GFP antibody to analyze the expression of GFP::DNC-2 and AIR-2::GFP. α-Tubulin was included as a loading control. Error bars are SD. The significance of the difference between results was determined by two-tailed *t* tests (ns, not significant difference; ****P < 0.0001). Asterisk denotes the target protein detected by the antibody. Source data are available for this figure.

Nuclear disassembly is triggered by phosphorylation of lamin at multiple residues in the head and tail domain by PLK-1 in *C. elegans* (Chase et al, 2000; Rahman et al, 2015; Velez-Aguilera et al, 2020). Therefore, we generated a strain-expressing PLK-1::GFP in *spd-3(oj35)* mutant to monitor the localization and expression of PLK-1. To our surprise, this strain revealed excessive amounts of PLK-1 in the *spd-3(oj35)* mutant localizing to centrosomes, the nuclear envelope, nucleoplasm, and chromosomes (Fig 5A and C and Video 8). We further confirmed that PLK-1 is increased by around sixfold in the *spd-3(oj35)* mutant compared with N2 animals by immunoblot (Fig 5B). However, the mRNA level of *plk-1* was not changed in *spd-3(oj35)* as assessed by RT-qPCR (Fig S8A). To test whether *spd-3(oj35)* mutants have generally increased gene expression, we tested the levels of Aurora B (AIR2) (Schumacher et al, 1998; Shao et al, 2015; Ali & Stukenberg, 2023), another mitotic kinase, and the dynein co-factor dynactin (DNC-2), which was previously suggested to be affected in the *spd-3(oj35)* (Dinkelmann et al, 2007). We found that in contrast to PLK-1 none of those two proteins was up-regulated (Fig S9B and C). Although we could not detect a significant difference in AIR-2 levels (*P* = 0.0664), all measured protein levels of AIR-2 in the *spd-3(oj35)* were below the levels detected for control embryos, suggesting some potential effect on AIR-2 levels (Fig S9B). Interestingly, DNC-2 showed lower protein levels in Western blots (Fig S9C), in contrast to previously reported results, which were based on measurements of fluorescence intensity. Overall, these results suggest that *spd-3* mutant embryos do not have globally increased gene expression. But it is possible that other proteins, in addition to PLK-1 and DNC-2, are affected in the *spd-3(oj35)* mutant. Interestingly, the decrease in DNC-2 levels could be involved in the observed spindle positioning defect, as has been shown for dynein depletion (Skop & White, 1998).

Video 8 Live-cell imaging of WT and *spd-3(oj35)* expressing PLK-1::GFP embryos during mitosis. The WT (left) and *spd-3(oj35)* (right) embryos expressing PLK-1::GFP. Fluorescence confocal images were acquired every 10 s by collecting 31 z-planes at 0.5-μm intervals. The images were processed by maximum projection. Sale bar represents 10 μm.Download video

### Reduction of PLK-1 levels prevents the SDC phenotype

In *C. elegans*, PLK-1 is recruited to the nuclear pore complexes (NPC) by nucleoporins NPP-1, NPP-4, and NPP-11 before NEBD where the importins α/β IMA-2 and IMB-1 promote the nuclear import of PLK-1, where it phosphorylates nuclear lamin (Martino et al, 2017). In addition, previous work showed that *ima-2 (RNAi)* is essential for spindle assembly and nuclear envelope formation in the *C. elegans* embryo (Askjaer et al, 2002). To determine the time point of PLK-1 import into the nucleus, we quantified the nuclear fluorescence of PLK-1::GFP in nucleoplasm during mitosis. The intensity of PLK-1::GFP reaches a peak about 2 min before anaphase onset in both pronuclei of control embryos (Fig 5C). This peak is shifted in *spd-3(oj35)* embryos to about 3 min before anaphase onset. In addition, we detected an overall increased fluorescence of PLK-1::GFP in the paternal pronucleus relative to the maternal pronucleus (Fig 5D). This result is consistent with the observed asymmetric and faster lamin disassembly in the paternal pronucleus (Fig 4B and D). In addition to the nucleoplasm, more PLK-1::GFP localizes to centrosomes. Centrosomes consist of a centriole pair surrounded by pericentriolar material (PCM) that nucleates and anchors microtubules (MTs) in *C. elegans*. In general, the centriole pair splits 53.8 ± 10.5 s after anaphase onset during mitosis (Fig 5E and F). Surprisingly, the centriole pair splits 126.4 ± 82.1 s before anaphase onset in *spd-3(oj35)* embryos (Fig 5E and F). This result is consistent with overexpression and inhibition of PLK1-affecting centriolar disengagement (Lončarek et al, 2010; Schöckel et al, 2011; Fu et al, 2015). These data indicate that overexpression of PLK-1 could lead to premature centriolar splitting, independent of the cell cycle regulation.

**Figure S10.**
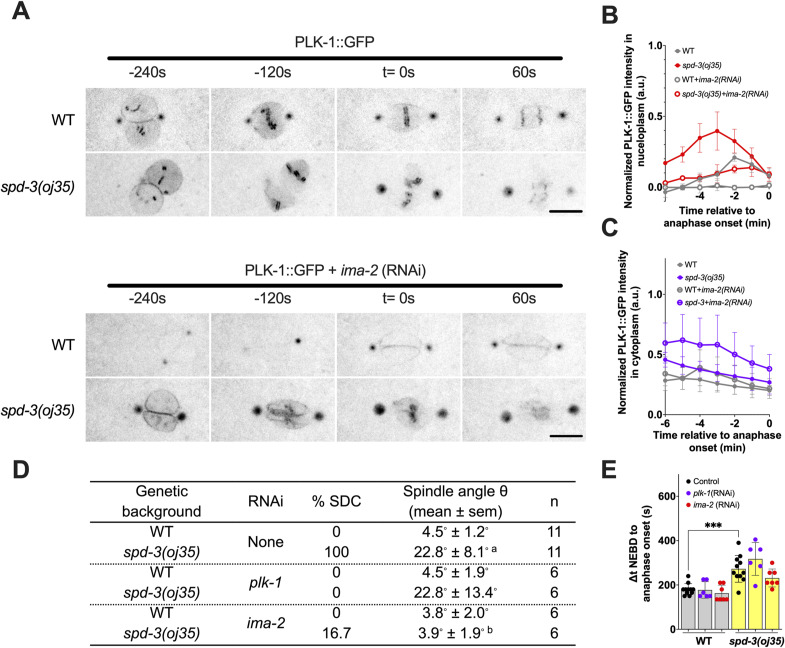
Reduction of PLK-1 in nucleoplasm can prevent the socially distanced chromosome positioning and spindle misalignment, related to Fig 6. **(A)** Time-lapse sequences of embryos expressing GFP::PLK-1 in WT and *spd-3(oj35)* treated without (top, processed by maximum projection) and with *ima-2 (RNAi)* (bottom). Times are in seconds relative to anaphase onset (t = 0 s). Scale bars, 10 μm. **(A, B)** Quantitation of normalized PLK-1::GFP intensity in the nucleoplasm of embryos from time-lapse sequences as in (A). The value of PLK-1 intensity in WT (gray, n = 6 for each condition) and *spd-3(oj35)* (red, n = 6 for each condition) without (solid) and with (empty) *ima-2(RNAi)*. **(A, C)** Quantitation of normalized PLK-1::GFP intensity in the cytoplasm of embryos from time-lapse sequences as in (A). The value of PLK-1 intensity in WT (gray, n = 6 for each condition) and *spd-3(oj35)* (purple, n = 6 for each condition) without (solid) and with (empty) *ima-2(RNAi)*. **(D)** Table of the percentage of socially distanced chromosomes phenotype and spindle orientation angle in WT and *spd-3(oj35)* embryos treated without or with *plk-1* (RNAi) or *ima-2* (RNAi). The “a” indicates *P < 0.05 in comparison to WT. The “b” indicates *P < 0.05 in comparison to *spd-3(oj35)*. **(E)** Plot of prometaphase duration in WT (gray bar) and *spd-3(oj35)* (yellow bar) without (black dots) and with *plk-1(RNAi)* (purple dots) or *ima-2(RNAi)* (red dots) treatment. Times are in seconds relative to NEBD (t = 0 s). The significance of the difference between results was determined by two-tailed *t* tests (ns, not significant difference). **(B, C, E)** Error bars are SD.

To determine whether reducing PLK-1 in the nucleoplasm can prevent the SDCs, spindle misalignment, and the delay in anaphase onset, we exposed the *spd-3(oj35)* worms to *ima-2 (RNAi)* to prevent nuclear import of PLK-1. In control embryos *ima-2 (RNAi)* prevents the nuclear import of PLK-1 and thus its localization to kinetochores and chromatin (Martino et al, 2017) (Fig S10A). In *spd-3(oj35)* mutant embryos, PLK-1::GFP still localizes to nucleoplasm and chromatin after *ima-2 (RNAi)* in embryos (Fig S10A); however, the amount of PLK-1 is reduced in nucleoplasm and increased in cytoplasm (Fig S10C) and the peak of PLK-1 fluorescence is shifted to about 2 min before anaphase onset, similar to control embryos (Fig S10B). Depletion of IMA-2 did not prevent the delay in anaphase onset; however, it rescued the spindle-positioning defect (Fig S10E). Interestingly, the SDC-positioning phenotype is partially rescued by *ima-2 (RNAi)* supporting the hypothesis that this phenotype is induced by elevated amounts of PLK-1 in the nucleus (Fig S10D).

**Figure 6.**
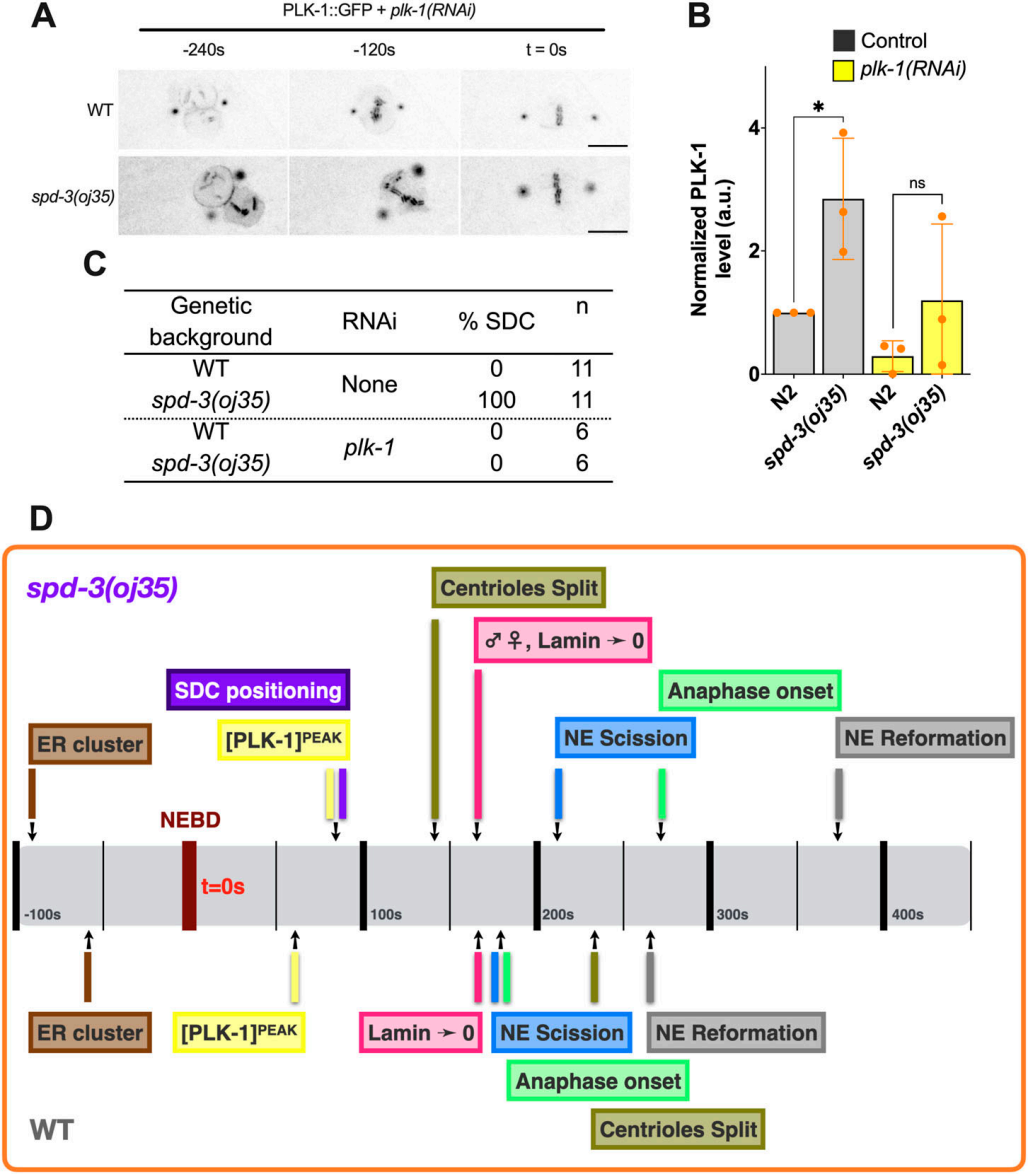
The socially distanced chromosomes phenotype in the *spd-3(oj35)* embryos results from PLK-1 overexpression. **(A)** Time-lapse sequences of embryos expressing PLK-1::GFP in WT (top) and *spd-3(oj35)* (bottom) after *plk-1(RNAi)* were filmed using a spinning-disk confocal. Times are in seconds relative to anaphase onset (t = 0 s). Scale bar, 10 μm. **(B)** Graph of the quantification of PLK-1 expression level in worm lysate from N2 and *spd-3(oj35)* without (gray bars, n = 3 for each strain) and with *plk-1(RNAi)* (yellow bars, n = 3 for each strain) as determined by Western blot. The significance of differences between results was determined by two-tailed *t* tests (*P < 0.05; ns, no significant difference). **(C)** Table of the percentage of socially distanced chromosomes phenotype in WT and *spd-3(oj35)* without and with *plk-1(RNAi)* or *ima-2(RNAi)* treatment. **(D)** Schematic showing the timeline of mitotic events in WT (bottom) and *spd-3(oj35)* (top).

To test this hypothesis, we exposed the *spd-3(oj35)* worms to short treatments of *plk-1 (RNAi)* to reduce the elevated levels of PLK-1 to approximately WT levels (Fig 6A and B).

Similar to IMA-2 depletion, reduction of the PLK-1 levels did not rescue the increased duration in time from NEBD to anaphase onset, but prevented spindle positioning and the SDC positioning (Fig 6C).

**Figure S11.**
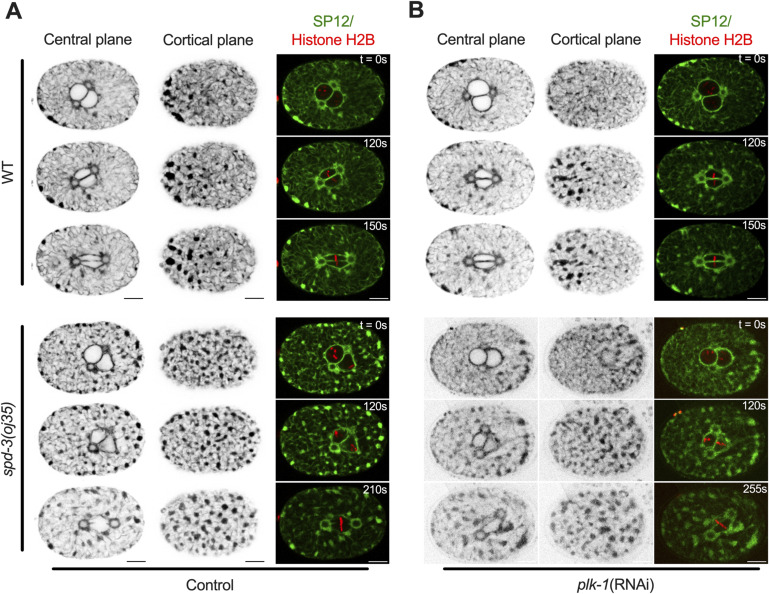
Changes in ER morphology are not induced by elevated PLK-1 levels, related to Fig 6. **(A, B)** Representative images of WT (top) and *spd-3(oj35)* (bottom) embryos treated without (A) and with (B) *plk-1*(*RNAi*) coexpressing SP12::GFP and mCherry::histone H2B. Images of the ER marker SP12 acquired at the central (left) and cortical plane (middle) and a merge of SP12 and histone H2B (right) are shown. Times are in seconds relative to NEBD (t = 0). Scale bars, 10 μm.

To determine if the abnormal ER morphology is also triggered by PLK-1 overexpression, we examined the ER structure after *plk-1 (RNAi)*. We found that the ER morphology and distribution of ER clusters are not different between control and *plk-1 (RNAi)* in *spd-3(oj35)* embryos, suggesting that the changes in ER morphology are not induced by elevated PLK-1 levels (Fig S11A and B). Our finding is thus consistent with the result that the removal of ER clusters cannot rescue SDCs (Fig S5).

## Discussion

Taken together we show that defects in SPD-3 cause a spindle positioning defect, an unusual chromosome-positioning (SDC) phenotype, changes in the ER structure and a dysregulation of mitotic events in *spd-3(oj35)* embryos (Fig 6D).

Our results revealed an increase in PLK-1 levels in the *spd-3(oj35)* embryos. PLK-1 is a master regulator in mitosis with multiple important roles to ensure the proper progression through mitosis such as chromosome alignment (Addis Jones et al, 2019), anaphase entry (von Schubert et al, 2015; O’Connor et al, 2015; Jia et al, 2016), and cytokinesis (Burkard et al, 2007; Rezig et al, 2021). In *C. elegans* PLK-1 was shown to be involved in establishment of polarity (Koch & Rose, 2023), centrosome maturation (Ohta et al, 2021; Nakajo et al, 2022), nuclear lamin disassembly (Velez-Aguilera et al, 2020), and disassembly of the nuclear pore complex (Nkoula et al, 2023). In agreement with the multiple roles of PLK-1, the *spd-3(oj35)* embryos show a number of defects that could be linked to PLK-1 overexpression.

The involvement of PLK1 in the regulation of the SAC is well established. PLK1, together with MPS1, phosphorylates several proteins involved in the establishment and maintenance of the SAC, such as Bub1, KNL1, and Mad1 and CDC20 (von Schubert et al, 2015; Jia et al, 2016). This phosphorylation promotes recruitment and retention of those SAC proteins at unattached or improperly attached kinetochores. Consequently, the SAC proteins are able to activate the checkpoint in response to unattached kinetochores, preventing premature anaphase onset. In addition, it has been observed that PLK1 also plays a role in stabilizing the kinetochore-microtubule attachment during prometaphase (Liu et al, 2012).

In light of our own experimental findings, we have noted that *spd-3(oj35)* embryos exhibit an extended duration of the time between NEBD and anaphase onset. However, this prolonged duration does not appear to be dependent on the presence of SAC proteins. All *spd-3(oj35)* embryos showed the SDC phenotype and with this a delay in metaphase plate formation with and without SAC proteins (Mad3 and Mad2). Interestingly, our data showed that chromosome and spindle positioning defects were elevated in embryos depleted of SAC proteins (Mad3 and Mad2) and often anaphase was initiated before the formation of a metaphase plate. Conversely, in untreated *spd-3(oj35)* embryos, with an intact SAC, anaphase onset only happened after a metaphase plate was established. These data suggest that the SAC contributes to reduce the chromosome and spindle alignment defects in the *spd-3(oj35)* embryos. Interestingly, it was previously shown that in *C. elegans*, SAC components like SAN-1 and MDF-2 are vital for preventing errors in cell division under extreme stress conditions, that is, anoxia (Nystul et al, 2003). Under oxygen-deprived conditions, embryos lacking SAN-1 or MDF-2 function show increased rates of chromosome missegregation, emphasizing a role for the SAC in preventing cells from dividing incorrectly when they have low energy. It remains unclear how the SAC proteins increase the fidelity of chromosome and spindle alignment defects in the *spd-3(oj35)* embryos and further research will be required to address this question.

PLK1 was also suggested to play a role in stabilizing the kinetochore–microtubule attachment during prometaphase and it is possible that the elevated levels of PLK-1 *in spd-3(oj35)* embryos could lead to the SDC phenotype affecting kinetochore-microtubule attachments and with this metaphase plate formation. In agreement with this, our data show that reducing PLK-1 levels indeed prevented the SDC phenotype, whereas we were not able to rescue the delay in anaphase onset. This could be due to difficulties reducing PLK-1 levels in the *spd-3(oj35)* embryos to WT levels. In addition, because PLK-1 is a key regulator of mitosis, already small deviations from the regular levels have major effects on mitosis. It is however also possible that the anaphase delay is independent of PLK-1 levels and rather a consequence of SAC activation.

Along this line we detected a decrease in the duration in the time between NEBD to anaphase onset of *spd-3(oj35)* after *rab-5(RNAi)* and *yop-1(RNAi)*. It was previously shown that RAB-5 and YOP-1, together with RET-1 are involved in ER structure and promoting nuclear envelope disassembly. Depletion of either RAB-5 or YOP-1/RET-1 leads to a delay in NEBD (Audhya et al, 2007). As we quantify the duration between NEBD and anaphase onset it is possible that a delay in NEBD upon *rab-5* and *yop-1(RNAi)* could shorten the overall duration between NEBD and anaphase onset in *spd-3(oj35)* to WT levels. It is however also possible that the extended timing between NEBD and anaphase onset in *spd-3(oj35)* is a consequence of disrupting the ER structure, as it has been suggested that the structure of the ER could potentially affect nuclear disassembly (Audhya et al, 2007).

PLK-1 plays a direct role in lamin disassembly, by phosphorylating lamin and thus targeting it for degradation (Velez-Aguilera et al, 2020). Our data showed that the paternal pronucleus disassembled several seconds before the maternal pronucleus. As PLK-1 localizes to centrosomes, which are initially associated with the male pronucleus this indicates that the asymmetric disassembly of the two pronuclei could be linked to excessive levels of PLK-1. Similarly, the chromosome alignment defects and faster lamin disassembly in *spd-3(oj35)*, and the reported reduction in mobility of SUN-1 aggregates and pairing-center binding proteins on the nuclear envelope in *spd-3(me85)* mutant (Labrador et al, 2013) could be linked to changes in PLK-1 levels.

SUN1 is a connector between the cytoskeleton and the chromosomes during pairing and alignment of homologous chromosomes during meiosis. PLK-1 phosphorylates SUN1, which regulates its function and localization (Labella et al, 2011; Patel et al, 2014). In particular, it facilitates the reorganization of SUN1 and its association with other proteins involved in chromosome pairing, such as ZYG-12. Thus, it is possible that elevated PLK-1 levels could lead to phosphorylation of SUN-1, affecting its mobility on the nuclear envelope. In addition, it was also shown that a complex of SUN1 and KASH is involved in mediating LINC complex–dependent nuclear movement and positioning (Cain et al, 2018). Therefore, it is possible that misregulation of SUN1 by elevated PLK-1 levels could also affect nuclear movement and spindle positioning. However, we found that DNC-2, a dynein adapter protein, is decreased in *spd-3(oj35)* (Fig S8C). A decrease in DNC-2 has previously been shown to result in spindle-positioning defects (Skop & White, 1998), which could possibly explain the observed spindle-positioning defect in *spd-3(oj35)*.

In addition, we also found spindle alignment defect phenotypes in embryos after RNAi of *ucr-1 (RNAi)*, *mev-1 (RNAi)*, and *cco-1 (RNAi)*, which alter the ETC function, resulting in decreased ATP levels, suggesting that mitochondrial ETC pathways could also affect mitotic spindle alignment, possibly even by affecting motor proteins or other energy-dependent processes (Table S1).

The remaining question is how the mitochondrial protein SPD-3 affects or regulates PLK-1 levels during mitosis in *C. elegans*. At this point, we can only speculate, but possible mechanisms include, that is, post-translational modifications (Macůrek et al, 2008) or protein stability (Cordeiro et al, 2020). Furthermore, detailed analysis will be necessary to determine the mechanisms and pathways used by SPD-3 that led to increased PLK-1 levels.

In summary, our work provides a novel link between mitochondria, progression through mitosis, nuclear envelope dynamics, and chromosome positioning by increasing the amount of a key mitotic regulator, PLK-1. This finding does not only provide a new role of the mitochondrial protein SPD-3 during mitosis in *C. elegans* but may also have further implications in the context of cancers or age-related diseases and infertility as it provides a novel link between mitochondria and mitosis and some insights into the effects of elevated PLK1 levels.

## Materials and Methods

### *C. elegans* strains and alleles

The Bristol strain N2 was used as the standard WT strain. Culturing, handling, and genetic manipulation of *C. elegans* were performed using standard procedures (Brenner, 1974). All *C. elegans* strains and the temperature-sensitive strain *spd-3(oj35)* were maintained at 16°C, and L4 hermaphrodites were shifted to RT (∼23°C) overnight and heat-shocked at 25°C for 4 h before analysis. *C. elegans* strains and RNAi feeding clones are listed in the Supplemental Material. L4 or young adult hermaphrodites were fed with RNAi bacteria and incubated for 6–24 h at RT (23°C) before dissection to obtain embryos for filming. The following alleles and strains were used: LGIII: *clk-1(qm30)*; LGIV: *isp-1(qm150)*, *spd-3(oj35)*. The *C. elegans* strains used in the study, carrying integrated fluorescent proteins, are outlined in Table S2. They were backcrossed four times with WT animals before being used for genetic analyses.

Table S2. The *C. elegans* strains carrying integrated GFP or mCherry used in this study are listed in the table above (Toya et al, 2010; Cheerambathur et al, 2019; Sanchez et al, 2021)

### RNA-mediated interference

The *ucr-1*, *mev-1*, *cco-1*, *yop-1*, *hsp-3*, *hsp-4*, *ima-2*, *mdf-2*, *plk-1*, *rab-5*, *san-1*, and *smg-1 (RNAi)* were administered by feeding. Feeding vector (L4440) was obtained from the Addgene. The *ucr-1*, *mev-1*, and *cco-1* RNAi-feeding clones were a gift from Orna Cohen-Fix laboratory (Maheshwari et al, 2021); the *rab-5*, *yop-1*, *hsp-3*, *hsp-4* RNAi-feeding clones were bought from Source Bioscience; the *mdf-2* RNAi-feeding clone was bought from Horizon Discovery. The *ima-2*, *plk-1*, *san-1*, and *smg-1* RNAi feeding vectors were constructed by cloning the *ima-2*, *plk-1*, *san-1*, and *smg-1* genomic DNA into the L4440 feeding vector (Table S3), followed by transformation into *Escherichia coli HT115(DE3)* bacteria. The *E. coli HT115(DE3)* bacteria containing the feeding vectors were cultured and used to seed RNAi plates (1 mM IPTG and 50 μg/ml ampicillin). L4 hermaphrodites were allowed to feed for 24 or 48 h at RT (∼23°C) and then shifted to 25°C for 4 h before analysis. For *plk-1* (RNAi), L4 larvae were cultured at RT overnight and then transferred to *plk-1 (RNAi)* feeding plates allowed to feed for 6 h at 25°C before analysis.

Table S3. Primers for *C. elegans* RNAi-feeding clones in this study.

### Live imaging

Embryos for live-imaging experiments were obtained by dissecting gravid adult hermaphrodites in M9 buffer (42 mM Na_2_HPO_4_, 22 mM KH_2_PO_4_, 86 mM NaCl, and 1 mM MgSO_4_). One-cell embryos were mounted on slides with 2% agarose pad, overlaid with a 22 × 22-mm coverslip, and imaged at RT on a wide-field fluorescence microscope (ECLIPSE Ti2; Nikon) equipped with a CFI Apo TIRF 60 × 1.45 NA oil immersion lens and a CCD camera (iXon 897 Ultra EMCCDs) or on a 3i VIVO spinning-disc confocal microscope (Axio Examiner.Z1; Zeiss) equipped with Zeiss Plan-Apochromat 63x/1.40 oil microscope objective, six laser lines and a Hamamatsu ORCA-Flash4.0 scientific CMOS camera (Hamamatsu) for detection. The microscope was controlled by Nikon NIS-Elements software (Nikon). For z-stacks, images in a 27 μm z-series (1 μm per section) were captured every 15 s (Figs 1A and B and S2; Table S1). Fluorescence confocal images were acquired every 10 s (Figs 4 and 5 and S9A–C; Video 8) or 15 s (Figs 1D, 2, and 3, S2D and F, S5, S7, S9E, S10, and S11; Video 1, Video 2, and Video 3) by collecting 31 z-planes at 0.5 μm intervals or 17 z-planes at 1 μm intervals without binning. Imaging was initiated in one-cell embryos before pronuclear meeting and was terminated 3 min after anaphase onset. EBP-2::GFP movies acquired with spinning-disc confocal microscope. Image processing was carried out with ImageJ software (images acquired at 400 ms intervals). Acquisition parameters were controlled using a SlideBook 6.0 program (3i—Intelligent Imaging). Each movie was 150 frames = 1 min (Fig S3; Video 3) (Srayko et al, 2005).

### Image analysis

All images were processed and analyzed using ImageJ (National Institutes of Health). For figure construction, final image panels were scaled for presentation in Prism v9.5.0 (GraphPad).

The angle of spindle orientation (θ) at the onset of anaphase in the one-cell stage of *C. elegans* embryos was determined using ImageJ. The spindle orientation angle refers to the orientation of the mitotic spindle relative to the anterior/posterior (A/P) axis of the embryo.

The width of the metaphase plate was determined by measuring the distance from top end to bottom end at the metaphase plate (perpendicular to the spindle axis) just prior to anaphase onset. For the quantification of distance between chromosomes, we measured the distance between the center of chromosome mass in the male and female pronucleus at time point after NEBD. Once the longest distance was analyzed, this stage was called “SDCs” positioning in *spd-3(oj35)* mutant.

The extend of ER cluster formation was assessed visually. Nuclear lamin fluorescence intensity (GFP::LMN-1) was quantified using ImageJ software by drawing a box around the nuclei and subtracting the background fluorescence in an equal-sized box drawn in the cytoplasm (Fig 3C and D). The total fluorescence intensity of nuclear GFP::LMN-1 was measured at each time point during mitosis. The mean value for this measurement is plotted versus time.

To quantify the PLK-1::GFP fluorescence in the nucleoplasm, a fixed-size box was drawn in nucleoplasm (red box) and in cytoplasm (black box) as background at each time point. The normalized PLK-1 level was calculated as ([integrated intensity in red box—integrated intensity in black box]/integrated intensity in black box) (Figs 4C and D and S7B). To quantify the PLK-1::GFP fluorescence in cytoplasm, a fixed-size box was drawn in cytoplasm and an area outside embryo as background at each time point. The normalized PLK-1 level was calculated as ([integrated intensity in red box—integrated intensity in black box]/integrated intensity in black box) (Fig S7C).

A gamma of 1.5 was applied for images of centrosomal PLK-1::GFP (Fig 4E) to visualize centrioles in the mutants, whereas not oversaturating the signal in the WT centrosomes.

The fluorescence of *hsp-4::ghp* reporter was measured using ImageJ software by drawing a box around the individual worm for quantification and subtracting the background fluorescence in an equal-sized box drawn over the background fluorescence (Fig S6).

For quantifications of centrosome size, centrosomes were marked with mCherry::γ-tubulin and line scans from single confocal planes over centrosome regions were used to quantify the centrosomes diameter and the full width at half maximum taken as centrosome diameter (Fig S2D).

For nucleation assays (Fig S3B), a half-circle (29–30 μm) was drawn around the centrosome, positioned 9 μm from the centrosome (as described in Sryako et al, 2005), and a kymograph of the entire movie (150 frames, 1 min) was generated by ImageJ. EBP-2::GFP comets on the kymograph were counted manually.

For measuring growth rates of cytoplasmic MTs (Fig S3D), we quantified EBP-2::GFP comets that could be observed for at least 10 frames for each embryo. We determined their start and stop positions within this period, and traced their paths manually using ImageJ to estimate their velocity.

### FLIM

A Zeiss LSM-780 NLO confocal/multiphoton microscopy system consisting of an inverted Axio Observer (Zeiss) microscope, motorized stage for automated scanning, Chameleon Vision-II (Coherent Inc.) ultrafast Ti:sapphire laser with dispersion compensation to maintain pulses at the specimen plane (690–1,060 nm, 80 MHz, 150 fs) for multiphoton excitation and a standard set of dry and immersion objectives was used. Three HPM—100-40 hybrid GaAsP detectors (Becker and Hickl) are connected to the nondescanned (NDD) port of the microscope using two T-adapters (Zeiss) with proper dichroics and band pass filters to collect as much fluorescence as possible in the spectral ranges tryptophan channel: 740 nm Ex, 340–380 nm Em, NAD(P)H channel: 740 nm Ex, 460–500 nm Em, and FAD channel: 890 nm Ex, 520–560 nm Em. The three channels also contain a 690 nm short pass filter (Zeiss) in the beam path to avoid excitation background. Three SPC-150 cards (Becker and Hickl) synchronized with the pulsed laser and the Zeiss LSM-780 scan head signals collect the time-resolved fluorescence in TCSPC mode using SPCM (Version 9.74) acquisition software. A motorized stage is used during imaging using Zeiss 40× NA1.3 oil apochromatic objective lens.

#### FLIM processing and analysis

FLIM data fitting is based on the B&H handbook. We used a two components incomplete model to fit tryptophan, NAD(P)H and FAD/FMN FLIM images. The offset and scattering are set to 0. Shift is optimized to make sure the Chi2 as close as to 1.

The FLIM processing followed the previously published article (Wallrabe et al, 2018; Cao et al, 2019) with an important advance in normalization of photon reference images to compensate for varying intensities (FIJI, Plugins—>Integral Image Filters—>Normalize Local Contrast): followed by zero’ing the nucleus; cell segmentation, and creating single pixel ROIs by a ImageJ/FIJI custom plugin. The purpose of this sequence is to create pixel locations by X–Y coordinates, specific for embryos. Those locations are then applied to the FLIM data to extract any of the FLIM parameters in the data pool. A custom Python code ultimately analyzes different data combinations to produce ratios, means, medians, and histograms, further charted in MS Excel.

### Immunoblotting

200 adult worms from each strain were transferred to 30 μl of sample buffer (80.0 mM Tris, pH 6.8, 2.0% SDS, 10.0% glycerol, 0.0006% bromophenol blue, and 5.0% β-mercaptoethanol), subjected to the freeze-thaw treatment three times in liquid nitrogen and water bath and then boiled in a 95°C-heating block for 5 min. The samples were loaded on 10% SDS–PAGE, transferred to a polyvinylidene fluoride (PVDF) membrane and subjected to Western blot analysis using anti-PLK-1 (1:3,000 dilution, gift from Monica Gotta laboratory, Tavernier et al [2015]) and anti-GFP (1:3,000 dilution, ab6556; Abcam). Primary antibodies were detected using IRDye 680RD goat anti-rabbit (1:5,000 dilution; ab21677; Abcam) and then probed for α-tubulin using the monoclonal DM1α antibody (1:3,000 dilution; ab7291; Abcam) and IRDye 800RD goat anti-mouse (1:5,000 dilution; ab21677; Abcam) to examine the expression levels of α-tubulin as a loading control. Antibody signals were detected using the Azure Biosystems c600 and Li-COR Fc Odyssey imaging system. The pixel intensity of protein bands was then quantified with Image J (http://rsbweb.nih.gov/ij/) and normalized the signal to signal of **α**-tubulin.

### Statistical analysis

Statistical analysis was conducted using Prism v9.5.0 (GraphPad). *P* values were determined using unpaired two-tailed *t* tests assuming equal SD. *P* > 0.05 (ns), *P* < 0.05 (*), *P* < 0.01 (**), *P* < 0.001 (***), and *P* < 0.0001 (****). Data distribution was assumed to be normal, but this was not formally tested.

## Supplementary Material

Reviewer comments
