# Supplementary material for: Loss of the mitochondrial protein SPD-3 elevates PLK-1 levels and dysregulates mitotic events
Source: Life Sci Alliance. 2023 Sep 8;6(11):e202302011. doi: 10.26508/lsa.202302011 (PMC10488725; doi:10.26508/lsa.202302011)
Supplement: Supplementary file 5 [file LSA-2023-02011_TableS1.docx]

| Genetic background/ RNAi | % SDC positioning | Spindle angle (θ)  (mean ± sem) | % Abnormal  nuclear shape | Prometaphase duration (sec) | n |
| --- | --- | --- | --- | --- | --- |
| WT (control) | 0 | 4.5^◦^ ± 1.2^◦^ | 0 | 180.0 ± 26.0 | 11 |
| *spd-3(oj35)* | 100 | 22.8^◦^ ± 8.1^◦ a^ | 100 | 272.7 ± 60.3^#^ | 11 |
| *ucr-1*(RNAi) | 0 | 11.8^◦^ ± 5.4^◦^ | 0 | 157.0 ± 12.1^✚^ | 3 |
| *mev-1*(RNAi) | 0 | 14,7^◦^ ± 0.9^◦^ | 0 | 195.0 ± 84.9^✚^ | 2 |
| *isp-1(qm150)* | 0 | 4.3^◦^ ± 1.3^◦^ | N.A. | 157.5 ± 23.7^✚^ | 10 |
| *cco-1*(RNAi) | 0 | 15.1^◦^ ± 3.8^◦^ | 0 | 200.0 ± 62.4^✚^ | 3 |
| *clk-1(qm30)* | 0 | 4.7^◦^ ± 1.3^◦^ | N.A. | 141.0 ± 16.1^✚^ | 10 |

**Table S1. Strains and RNAi feeding clones for the electron transfer chain (ETC) pathway in mitochondria, related to Figure 1.**

^a^ One-way ANOVA was used for statistical analysis (**P* < 0.05). ^#^ One-way ANOVA was used for statistical analysis (*****p* < 0.0001). ^✚^ One-way ANOVA was used for statistical analysis (ns, not significant difference). N.A, not applicable.
