# Supplementary material for: Loss of the mitochondrial protein SPD-3 elevates PLK-1 levels and dysregulates mitotic events
Source: Life Sci Alliance. 2023 Sep 8;6(11):e202302011. doi: 10.26508/lsa.202302011 (PMC10488725; doi:10.26508/lsa.202302011)
Supplement: Supplementary file 11 [file LSA-2023-02011_TableS2.docx]

| Strain | Genotype | Reference |
| --- | --- | --- |
| WH342 | *unc-119(ed3) III; ojIs31* [P*_pie-1_*SPD-3::GFP + *unc-119(+)*] | Dinkelmann et al. 2007 |
| SA250 | *unc-119(ed3) III; tjIs54* [P*_pie-1_*GFP::TBB-2 + P*_pie-1_* 2xmCherry::TBG-1 + *unc-119(+)*]*;* *tjIs57* [P*_pie-1_* mCherry::HIS-48 + *unc-119(+)*] | Toya et al. 2010 |
| TV24458 | *wow47* [*ebp-2::gfp::3xflag)*] *II; zif-1(gk117) III; wyEx9745* | Sanchez et al. 2021 |
| SJ4005 | *zcIs4* [P*_hsp-4_*GFP] | Calfon et al. 2002 |
| WH327 | *unc-119(ed3) III; ojIs23* [P*_pie-1_*GFP::SP12] | Poteryaev et al. 2005 |
| SBW244 | *sbw8* [*ndc1(npp-22)::mNeoGreen*] | Mauro et al. 202 |
| UV117 | *jf98* [*gfp::lmn-1*] I | Link et al. 2018 |
| OD2425 | *lt18* [*plk-1::sgfp*]*::loxp* III | Martino et al. 2017 |
| WH258 | *unc-119(ed3) III; ojIs5* | Dinkelmann et al. 2007 |
| OD3230 | *air-2(lt58[air-2::GFP::tev::loxP::3xFlag]) I* | Cheerambathur et al. 2019 |

**Table S2. The *C. elegans* strains carrying integrated green fluorescent protein (GFP) or mCherry used in this study are listed in the table above.**

The *spd-3(oj35)* allele was outcrossed against N2 five times before crossing into fluorescent marker strains for phenotypic analysis.
