# Supplementary material for: Loss of the mitochondrial protein SPD-3 elevates PLK-1 levels and dysregulates mitotic events
Source: Life Sci Alliance. 2023 Sep 8;6(11):e202302011. doi: 10.26508/lsa.202302011 (PMC10488725; doi:10.26508/lsa.202302011)
Supplement: Supplementary file 12 [file LSA-2023-02011_TableS3.docx]

| Gene | Primer 1 | Primer 2 | Template |
| --- | --- | --- | --- |
| F26B1.3  *(ima-2)* | TATAGCGGCCGCCTGTC  TCACAATGCCGAAGA | ATAGCTAGCTGTCAGC  GAGTCATTTCCAG | N2 genomic DNA |
| C14B9.4  *(plk-1)* | TATAGCGGCCGCTCAAC  AACAAGCTGCAGAGG | ATAACGCGTTGGGACTA  AAAGGGTCGATG | N2 genomic DNA |
| *ZC328.4 (san-1)* | TGGCAGAAGAACCGTACGTTT | CAATGTCTCATCGAAATCCAACT | N2 genomic DNA |
| C48B6.6  *(smg-1)* | TATAGAGCTCAAAACCGCGATAATCGAATG | ATAGCGGCCGCccagcaagtgttgcttggta | N2 genomic DNA |

**Table S3. Primers for *C. elegans* RNAi feeding clones in this study.**
